# The DNA Methylome of Human Peripheral Blood Mononuclear Cells

**DOI:** 10.1371/journal.pbio.1000533

**Published:** 2010-11-09

**Authors:** Yingrui Li, Jingde Zhu, Geng Tian, Ning Li, Qibin Li, Mingzhi Ye, Hancheng Zheng, Jian Yu, Honglong Wu, Jihua Sun, Hongyu Zhang, Quan Chen, Ruibang Luo, Minfeng Chen, Yinghua He, Xin Jin, Qinghui Zhang, Chang Yu, Guangyu Zhou, Jinfeng Sun, Yebo Huang, Huisong Zheng, Hongzhi Cao, Xiaoyu Zhou, Shicheng Guo, Xueda Hu, Xin Li, Karsten Kristiansen, Lars Bolund, Jiujin Xu, Wen Wang, Huanming Yang, Jian Wang, Ruiqiang Li, Stephan Beck, Jun Wang, Xiuqing Zhang

**Affiliations:** 1 BGI-Shenzhen, Shenzhen, Guangdong, China; 2 Cancer Epigenetics and Gene Therapy Program, Shanghai Cancer Institute, Shanghai Jiaotong University, Shanghai, China; 3 Epigentic Laboratory, Bio-X Center, Shanghai JiaoTong University, Shanghai, China; 4 Beijing Institute of Genomics, Chinese Academy of Sciences, Beijing, China; 5 The Graduate University of Chinese Academy of Sciences, Beijing, China; 6 School of Bioscience and Biotechnology, South China University of Technology, Guangzhou, China; 7 Kunming Institute of Zoology, Chinese Academy of Sciences, Yunnan, China; 8 Department of Biology, University of Copenhagen, Copenhagen, Denmark; 9 Institute of Human Genetics, University of Aarhus, Aarhus, Denmark; 10 Institute of Genetics and Developmental Biology, Chinese Academy of Human Sciences, Beijing, China; 11 UCL Cancer Institute, University College London, London, United Kingdom; The Babraham Institute, United Kingdom

## Abstract

DNA methylation plays an important role in biological processes in human health and disease. Recent technological advances allow unbiased whole-genome DNA methylation (methylome) analysis to be carried out on human cells. Using whole-genome bisulfite sequencing at 24.7-fold coverage (12.3-fold per strand), we report a comprehensive (92.62%) methylome and analysis of the unique sequences in human peripheral blood mononuclear cells (PBMC) from the same Asian individual whose genome was deciphered in the YH project. PBMC constitute an important source for clinical blood tests world-wide. We found that 68.4% of CpG sites and <0.2% of non-CpG sites were methylated, demonstrating that non-CpG cytosine methylation is minor in human PBMC. Analysis of the PBMC methylome revealed a rich epigenomic landscape for 20 distinct genomic features, including regulatory, protein-coding, non-coding, RNA-coding, and repeat sequences. Integration of our methylome data with the YH genome sequence enabled a first comprehensive assessment of allele-specific methylation (ASM) between the two haploid methylomes of any individual and allowed the identification of 599 haploid differentially methylated regions (hDMRs) covering 287 genes. Of these, 76 genes had hDMRs within 2 kb of their transcriptional start sites of which >80% displayed allele-specific expression (ASE). These data demonstrate that ASM is a recurrent phenomenon and is highly correlated with ASE in human PBMCs. Together with recently reported similar studies, our study provides a comprehensive resource for future epigenomic research and confirms new sequencing technology as a paradigm for large-scale epigenomics studies.

## Abstract 2

Analysis across the genome of patterns of DNA methylation reveals a rich landscape of allele-specific epigenetic modification and consequent effects on allele-specific gene expression.

## Author Summary

Epigenetic modifications such as addition of methyl groups to cytosine in DNA play a role in regulating gene expression. To better understand these processes, knowledge of the methylation status of all cytosine bases in the genome (the methylome) is required. DNA methylation can differ between the two gene copies (alleles) in each cell. Such allele-specific methylation (ASM) can be due to parental origin of the alleles (imprinting), X chromosome inactivation in females, and other as yet unknown mechanisms. This may significantly alter the expression profile arising from different allele combinations in different individuals. Using advanced sequencing technology, we have determined the methylome of human peripheral blood mononuclear cells (PBMC). Importantly, the PBMC were obtained from the same male Han Chinese individual whose complete genome had previously been determined. This allowed us, for the first time, to study genome-wide differences in ASM. Our analysis shows that ASM in PBMC is higher than can be accounted for by regions known to undergo parent-of-origin imprinting and frequently (>80%) correlates with allele-specific expression (ASE) of the corresponding gene. In addition, our data reveal a rich landscape of epigenomic variation for 20 genomic features, including regulatory, coding, and non-coding sequences, and provide a valuable resource for future studies. Our work further establishes whole-genome sequencing as an efficient method for methylome analysis.

## Introduction

DNA methylation plays a vital role in genome dynamics. In the human genome, it predominantly occurs at cytosine guanine dinucleotide (CpG) sites in somatic cells [1] and at non-CpG cytosines in embryonic stem cells [2] and perhaps other cells as well. DNA methylation at any of these sites can vary and thus affect many biological processes that impact on human health and disease [3]. Therefore, detailed knowledge of the of DNA methylation status of all cytosines (the methylome) is paramount for understanding the mechanisms and functions underlying DNA methylation.

The emergence of the next-generation sequencing of bisulfite converted DNA represents an important advance in the field of DNA methylation analysis [4]–[6]. This technology has enabled human methylome analysis to advance from single chromosomes [7] to low (100 bp) resolution whole genomes [8] to single-base resolution whole genomes using bisulfite sequencing [2],[9]. For a comprehensive description of methylome analysis methods, please refer to the recent review by P. Laird [10].

Using whole-genome bisulfite sequencing, we here report the methylome analysis of peripheral blood mononuclear cells (PBMC) from an anonymous male Han Chinese individual (YanHuang) whose genome was determined in the first Asian genome project, henceforth referred to as YH [11]. This approach allowed us to analyse approximately 20 million CpG sites of this clinically important human methylome for genomic landscape, allele-specific methylation (ASM), and allele-specific expression (ASE) in primary cells in a single individual.

## Results

### Data Generation and Quality Assessment

The methylome reported and analyzed here was generated from the same sample of peripheral blood mononuclear cells (PBMCs) from a consented donor whose genome was deciphered in the YH project [11]. The nuclear DNA was extracted and subjected to unbiased, whole-genome bisulfite sequencing (BS-seq) using the Illumina Genome Analyzer (Table S1a) [5],[12]. In total, we generated 103.5 Gbp of paired-end sequence data. Of these, 70.4 Gbp (68%) were successfully aligned to either strand of the YH genome [11] with an average mismatch rate of 1.3% (Table S1b), resulting in an average sequencing depth of 12.3-fold per DNA strand or a 24.7-fold overall depth. Of the 18,962,679 CpGs present in the unique haploid part (2.21 Gb) of the YH reference genome sequence, approximately 99.86% were covered by at least one unambiguously mapped read of quality score >14 on either strand, and 92.62% were unambiguously covered on both strands (Figure S1 shows the cumulative distribution of sequencing depth; see Methods for details). Based on the 24.7-fold overall coverage, we estimated that about 88.1% of CpGs were covered on both alleles, but only 6.2% of CpGs could be definitively defined due to the limited number of nearby SNPs. We therefore only used these 6.2% (or 1.17 million) CpG sites for our allele-specific methylation analysis. Based on alignment to in silico converted non-CpG cytosines, the bisulfite conversion rate was determined to be at least 99.8% even assuming all non-CpG methylcytosines are due to conversion failure, ensuring reliable ascertainment of CpG methylcytosines at a false positive rate of <0.5%. All five libraries (Table S1a) showed similar conversion rates (99.7% to 99.9%), and a linear correlation was observed in methylation levels estimated from different libraries (Figure S2). This demonstrates high consistency between technical replicates. We also performed conventional bisulfite Sanger sequencing in randomly selected regions and found that 100% (50 of 50 tested CpG sites) showed a consistent methylation level (*p*>0.01 in chi-square test; Table S8). The rate of unconverted non-CpG cytosines is a combination of incomplete conversion and authentic non-CpG methylation, which indicates very low methylation levels (<0.2%) of non-CpG cytosines in PBMC. We also used the methylation ascertainment method based on binomial test and false discovery rate constraint that was applied by Lister et al. [2] to distinguish putative non-CpG methylation sites from incomplete bisulfite conversion and found a comparable (<0.2%) rate of non-CpG methylation in human PBMC. Non-CpG methylation roughly followed an exponential distribution where only a few (<1e−5) cytosines had methylation levels of >80% (Figure S3). We used these findings to exclude non-CpG methylation from subsequent analyses and estimate the overall specificity of identified methylcytosines in the PBMC methylome presented here to be 99.5%. We also used computing simulation to estimate the false negative rate of methylation site discovery. Assuming the methylation levels of CpG cytosines are similar between hESC [2] and PBMC, we estimate that about 13% of methylated CpG sites would be missed, of which a majority would be hypomethylation (<20%) sites. This indicates the PBMC methylome has a sensitivity to detect most methylated CpG sites.

### Landscape of PBMC Methylome

**Figure 1:**
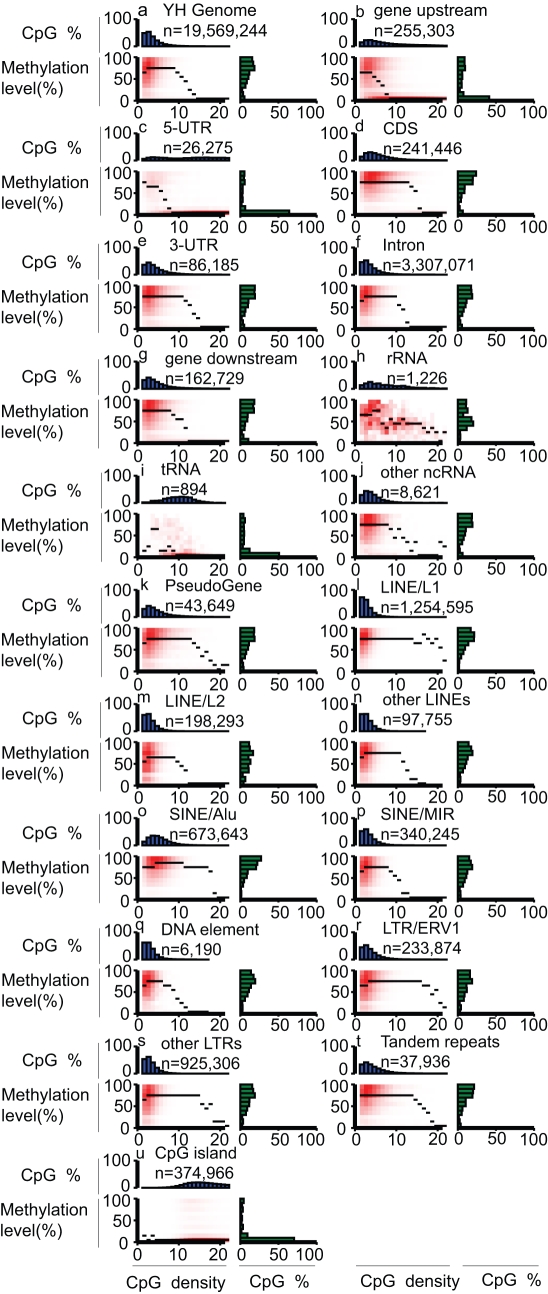
Landscape of the PBMC methylome. Heat maps show distinct methylation and CpG density patterns for different genomic features. Each panel represents a separate feature, and *n* refers to the number of analyzed CpGs (per-strand depth ≥10) within that feature. CpG density (*x-*axis) is defined as the number of CpG dinucleotides in 200 bp windows. Methylation level (*y*-axis) is defined as the mean methylation level of cytosines in CpGs. The thin black lines within each heat map denote the median methylation level of CpGs at the given local density. The red gradient indicates the abundance of CpGs that fall into bins of given methylation levels and CpG densities. The blue bar charts above each heat map show the distribution of CpG densities, projected onto the *x*-axis of the heat maps. The green bar charts to the right of the heat maps show the distribution of methylation levels, projected onto the *y*-axis of the heat maps.

We carried out a global analysis of the PBMC methylome and found the overall CpG methylation level to be 68.4%, which is lower than in H1 human embryonic stem cells (ESC) [2] but is still considered to be relatively high. Next, we determined the methylation distribution (Figure 1a) and showed it was less bimodal (9.27% hypo (<20%) methylated, 28.81% hyper (>80%) methylated) than has been previously observed (27.4% and 42.4%, respectively) [7], reflecting less bias of the whole-genome approach used here. Chromosome-specific effects could be excluded based on a separate analysis (Figure S4) of the three chromosomes analysed in the previous study [7]. Most notable was that the methylation distribution was not significantly affected by our depth threshold (where 4-fold was the lowest depth; Figure S10). In support of the conclusions drawn here, these data were consistent with the previous observation showing the CpG methylation level to peak at >70% in the human ESC methylome using the same bisulfite sequencing technology [2]. The whole genome CpG density showed a negative correlation with previously observed methylation levels [2],[6], while a major decrease was observed when CpG density rose from 10 to 15 per 200 bp windows.

**Figure 2:**
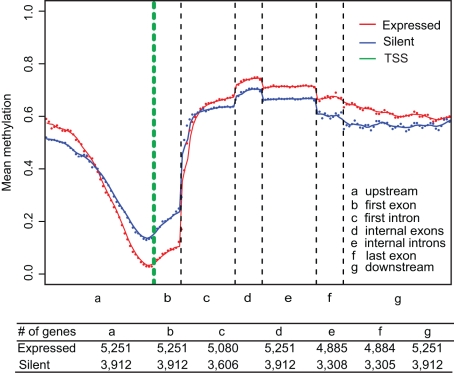
Canonical DNA methylation profiles of expressed and silent genes in PBMC. Expression status was determined by digital gene expression profiling (DGEP). Genes with ≥5 DGEP tags were defined as expressed (*n* = 5,251, color-coded red). Genes with no DGEP tag were defined as silent (*n* = 3,912, color-coded blue). The canonical gene structure is defined by 7 different features, denoted by the *x*-axis. The length of each feature was normalized and divided into equal numbers of bins. Each dot denotes the mean methylation level per bin and the respective lines denote the 5-bin moving average. Each feature was analyzed separately for the numbers listed in the table below the figure. The green vertical line indicates the mean location of the transcription start sites (TSS).

We next performed a comprehensive analysis of the PBMC methylome for an additional 20 distinct genomic features (Figure 1b–u). Although some of these features have been analysed before [2],[6],[7],[13] (reviewed in [13]), our analyses provided additional information as well as a more global assessment of some of these components. For example, with respect to protein-coding genes, our data enabled the integration of multiple features into higher-order structures, such as canonical DNA methylation profiles across the entire transcriptional units of expressed and silent genes (Figure 2). Up to 13% difference (*p*<1e−42) in methylation between highly expressed and silent genes (as determined by digital gene expression profiling (DGEP) of the same sample) are clearly visible, as are two discrete switchover zones, one upstream of the TSS and one in intron 1 that demarcates the transition from hypo- to hypermethylation in the inverse relationship between promoter and gene-body methylation and expression. Also evident is a distinct elevation in methylation level at internal exons with clear demarcation of intron/exon boundaries.

To define genes as expressed or silent, we grouped them according to their DGEP tags, allowing correlation to be assessed between averaged levels of DNA methylation and gene expression. However, other factors than DNA methylation can of course affect expression levels, and future analysis of samples from different tissues should help to address this issue. Within these limitations, we observed a clear trend for DNA methylation levels of expressed genes to decrease at TSS and to increase at gene bodies. This is consistent with results reported from bisulfite sequencing of human ESC [2].

For non-coding RNA genes, we found that different gene families had very different methylation profiles (Figure 1h–j). For instance, tRNA genes had a significantly (*p*<1e−343) lower methylation level than rRNA genes and the genome average. We further conducted a comprehensive analysis of repeat elements, which is a particular strength of our unbiased whole-genome BS-seq approach (Figure 1k–t). Here we found elements that were still active, such as long terminal repeats (LTRs), LINE/L1, and SINE/Alu, and had significantly higher methylation levels than genome average (*p*<1e−100), displaying hypermethylation even at high CpG density (>12 CpGs in 200 bp). For instance, we found that methylation levels in Alu elements negatively correlated with evolutionary sequence divergence (Figure S5) and thus negatively correlated with retrotransposon mobility [14]. Loss of methylation in such transposable elements is known to be associated with tumorigenesis [15],[16], and the above observations are consistent with DNA methylation playing a role in controlling retrotransposon mobility by lowering their activities and thereby stabilizing the genome.

In methylome studies, CpG islands are a special genomic feature of great interest (Figure 1u). To investigate these, we performed a canonical analysis of CpG islands and found CpG density and methylation levels displayed a mirrored pattern (Figure S11). CpG islands are CpG-rich and generally hypomethylated, and the shores [17] showed gradual transition of CpG density and methylation levels between the CpG islands and genome average.

### Co-Methylation

Next, we examined the correlation of methylation level of any two nearby CpGs and the relationship between spatial distance (from one CpG to another) and strength of this correlation. Gaining knowledge of genomic regions or features that are highly correlated in methylation status is advantageous for developing efficient designs for genome-wide association studies by enabling the selection of tag CpGs, analogous to tag SNPs [18]. As has been previously observed [7], co-methylation deteriorates over distance and becomes nearly undetectable at distances >1,000 bp (Figure S6a). The co-methylation observed here was not affected by the underlying CpG density (Figure S12).

Analysis of CpG cytosines that had the same distance between them showed that higher methylation levels correlate when they were located on the same strand than on opposite strands (*p<*6e−7; Figure S6a). This is presumably due to temporary hemi-methylation as a result of post-replication lag in methylation maintenance in proliferating cells. Co-methylation is also markedly different between different genomic features (Figure S6b–t). For example, the correlation was significantly (*p*<1e−30) higher in gene- than in repeat-associated features. Using Fourier transformation, we also tested the methylation correlation for patterns and found a significant (*p*<1e−4) peak in periodicity of approximately 170 bp (Figure S7). A similar (CHG) methylation pattern was observed in *Arabidopsis* [4], which the researchers suggested was due to a nucleosome positioning effect on co-methylation. However, no significant periodicity of smaller motifs was observed in our data.

### Tissue-Specific Differentially Methylated Regions

We compared the PBMC methylome to that of fetal lung fibroblast cells (IMR90) [2],[8] to assess potential tissue-specific differentially methylated regions (tDMR). In total, 240,856>200 bp independent regions (range 200–3.5 kbp; median size 500 kbp; see Methods for more details) that had significant differences in methylation level (>2-fold change, at least in one tissue is not hypomethylated (<20%) and Fisher test *p* value <1e−2) were identified as candidate tDMRs. Of these, 6,197 were located in the 2 kb flanking sequences of transcription start sites (TSSs) of 6,415 genes. GO classification showed that genes associated with PBMC-specific, hypomethylated tDMR candidates (and confirmed to be expressed according to DGEP analysis and/or the GEO database [19]) were significantly (*p*<1e−4) overrepresented in categories that related to DNA damage checkpoint (Tables S2, S3).

### Allele-Specific Methylation

**Figure 3:**
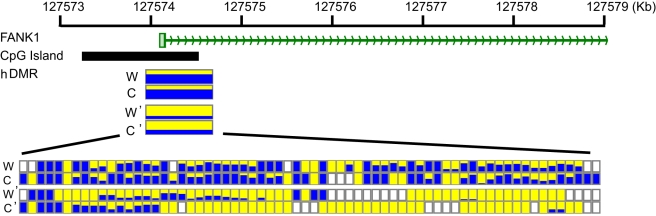
Example of a gene (*FANK1*) newly identified to display allele-specific methylation (ASM). Tracks 1 and 2 show the position of exon 1 and the associated CpG island, respectively. Track 3 shows the position of the identified haploid differentially methylated region (hDMR), W and C denote the forward (Watson) and reverse (Crick) strands of allele 1, and W' and C' denote the corresponding strands of allele 2. The DNA methylation status is color-coded: hypomethylated (yellow) and hypermethylated (blue). The bottom track shows the underlying bisulfite sequencing data for each CpG in the hDMR. The color code is as above, except for unfilled boxes, which denote the absence of data. The actual methylation level (shown as yellow:blue ratio) was derived from an average of 14.7 reads per CpG site.

We examined the PBMC methylome to assess allele-specific methylation (ASM) in the context of genomic imprinting [20] and allele-specific expression (ASE) [21]. Integration of our methylome data with the YH haploid genome sequences [11] enabled us to determine ASM for 1.17 million CpG sites (see above), providing an unprecedented opportunity to identify a first and comprehensive set of haploid differentially methylated regions (hDMR) in any human cell type. Using a conservative threshold (≥5 CpGs with at least 2-fold methylation difference and *p* value <0.001 in Fisher test), we identified 599 hDMRs (mean size of 312 bp), which accounted for 0.61% of all CpGs with biallelic methylation information or 0.33% of 181,599 regions with bi-allelic sequence information and ≥5 CpGs in their 300 bp flanking sequences (Table S4, see Methods for details). For each of the hDMRs, we randomly selected genomic CpGs with same sequencing depths to that of the hDMRs and subjected them to 10,000 bootstrap iterations to determine how many times the randomly selected CpGs would show differential methylation as defined above. The simulation indicated that 4.17% of these hDMRs were stochastic (showing hDMR signals in >5% of the simulations). As there are approximately 28 million CpG sites in the human genome and ASM could be ascertained for 1.14 million sites, we extrapolated the total number of hDMRs in the YH methylome to be approximately 10,000. This rate, however, is likely to be an overestimation because: (1) 300 bp windows with <5 CpGs may not have enough statistical power to distinguish ASM and (2) CpGs in such lower-CpG-density regions are generally hypermethylated and statistically less likely (likelihood ratio <0.133 compared to regions with ≥5 CpGs in 300 bp windows based on 100,000 simulations on the PBMC methylome) to qualify as ASM according to the conservative threshold described above (*p*<0.001, 2-fold methylation level change). Nonetheless, if none of the 300 bp windows with <5 CpGs were to display ASM, and those with ≥5 CpGs were to have the same rate of displaying ASM as observed in flanking regions with bi-allelic sequence information, the lower limit of the total number of hDMRs in the YH methylome would still be expected to be 5,000. Thus, we estimate that 0.3%–0.6% of the YH genome are subject to ASM. Annotation analysis revealed that some of these hDMRs were associated with 287 genes (see Table S5 for full list). Figure 3 shows an example of such an association (with the gene *FANK1*), which displays ASM. *FANK1* is a testis-specific gene and has been proposed to play a role in the transition from the diploid to the haploid phase during spermatogenesis [22]. In addition, we investigated the distribution of hDMRs within the YH genome. This analysis revealed a significant (*p*<1e−343) tendency for the hDMRs to cluster, particularly when in proximity to telomeres or centromeres, which are both hallmarks of imprinting.

**Figure 4:**
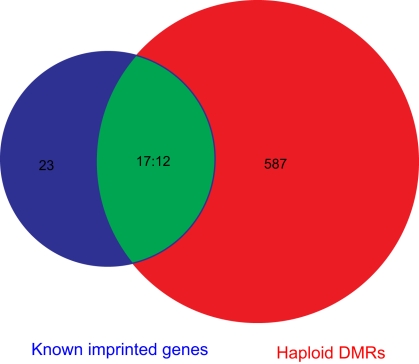
Venn diagram showing the relationship between haploid differentially methylated regions (hDMRs, red), known imprinted genes (blue), and their intersections (green). In the intersection, 17 known imprinted genes overlapped with 12 hDMRs in their genomic space.

To assess the potential of the hDMRs to denote known or novel imprinted loci, we tested the 599 identified hDMRs for correlation with known imprinted loci [23]. First, we analysed the known genomic imprinted space (defined by 40 loci in 15 chromosomal regions [23]) and identified 17 overlaps (Figure 4), including with well-known imprinted loci such as *IGF2*, *H19*, *KCNQ1*, *GNAS*, and others (Figure S8). Reciprocal analysis of known imprinted loci for which bi-allelic information was available showed 87.8% ASM, indicating that most of the ASM regions can be identified by bisulfite sequencing and that the major limiting factor is a lack of SNPs to differentiate the two alleles. We therefore estimate that most of the hDMRs are not attributable to imprinting but to other mechanisms such as sequence-dependent ASM [24].

Finally, we analysed the possible involvement of ASM (defined by presence of hDMRs) in epigenetically driven allele-specific expression (ASE) (reviewed in [21]). For this, we randomly selected 6 of the 76 genes that had one or more hDMR(s) within 2 kb of their TSS and measured their expression by TA clone sequencing. Five of the six genes (83%) showed a >1.5-fold difference in expression level between the two alleles (Table S6), confirming the inverse relationship between promoter ASM and ASE. As ASM is definitive for 6.2% of the genome and 76 genes had hDMRs within 2 kb flanking sequence, we estimated that 600 to 1,200 genes (3%–6%) display ASM, which indicates that up to a quarter of the 20% of human genes that have been reported to display ASE [25] may be driven by ASM.

To determine possible biological functions of the 76 genes displaying ASM, we carried out gene ontology (GO) analysis. Our results showed that these hDMR-containing genes are significantly (*p*<1e−4) overrepresented in function categories related to cell division and differentiation (see Table S7 for full list of significant GO categories), such as “negative regulation of S phase of mitotic cell cycle,” “mitotic metaphase/anaphase transition,” and “negative regulation of lymphocyte proliferation.” This functional enrichment pattern was also supported by hDMR-containing non-coding RNA genes *hY3* and *hY5* that were reported to be essential in DNA replication [26], which is an integral part of cell division.

## Discussion

In this study, we have generated and analysed the two haploid methylomes of human peripheral blood mononuclear cells (PBMC) from an individual whose genome was previously sequenced. This allowed, for the first time, for assessment of the level of ASM within a human methylome and extends recent studies analysing variation between different human methylomes [2],[9]. Compared to what was observed in embryonic stem cells in these studies, non-CpG methylation in human PBMC was negligible.

Our results show that ASM is more frequent than can be accounted for by known imprinted loci [23] and correlates very well with ASE for genes displaying ASM in their promoter regions. To further quantify this observation, additional methylomes will be required to increase the number of parental polymorphisms at imprinted regions. Nonetheless, our work provides a first proof-of-concept for the importance of including ASM in methylome analyses.

In addition, our data revealed a rich landscape of distinct epigenomic features for regulatory, coding, and non-coding sequences. Exons, for instance, were clearly discernable from introns by elevated methylation levels, demarcated by sharp intron-exon boundaries. This finding confirms and extends a recent observation that exons can be defined by epigenetic marks such as nucleosome positioning [13],[27]. The nature of our whole-genome approach enabled us to also analyse features that have previously been difficult to assess [28] such as repeat elements that constitute about 50% of the human genome [29]. Mobility of Alu repeat elements, for instance, was found to negatively correlate with their methylation levels, emphasizing the critical role of DNA methylation in genome stability.

In conclusion, we have reported the first comprehensive methylome analysis at single base-pair resolution for human blood cells with relevance to basic and clinical research. Our results demonstrate this methylome to be rich in biological information, compatible for integration with functional data, and we expected it to form a lasting resource as part of the International Human Epigenome Project [30].

## Materials and Methods

### Data Availability

The PBMC methylome data have been deposited into the NCBI Gene Expression Omnibus (http://www.ncbi.nlm.nih.gov/geo/query/acc.cgi?acc=GSE17972). In addition, the PBMC methylome and other data are available at the YH genome database (http://yh.genomics.org.cn).

### Public Data Used

The YH genome was downloaded from YH database (http://yh.genomics.org.cn). Gene and repeat annotations were downloaded from the UCSC database (http://genome.ucsc.edu/). The NCBI reference genes with prefix “NM” were mapped to the reference genome using BLAT by UCSC. Hits with >90% identity were retained for further analysis and only one transcript was retained for each gene. Known imprinted genes were extracted from the content of [23], respectively.

### Sample Preparation and Bisulfite Sequencing

Peripheral blood was obtained from the same individual as in the YH project, and mononuclear cells were separated through Ficoll-Paque (GE Heatlthcare) gradient centrifugation. The total DNA was prepared by proteinase K/phenol extraction, and RNA was extracted from mononuclear cells with RNeasy Mini Kit (Qiagen) following the manufacturer's instructions. The DNA was fragmented by sonication using a Bioruptor (Diagenode, Belgium) to a mean size of approximately 250 bp, followed by the blunt-ending, dA addition to 3′-end and, finally, adaptor addition (in this case of methylated adaptors to protect from bisulfite conversion), essentially according to the manufacturer's instructions. The bisulfite conversion of the adaptor-added DNA was carried out as previously described [31]. Raw GA sequencing data were processed by Illumina Pipeline v1.3.1.

Validation of the methylated state of selected candidate loci was performed by sequencing of multiple T-cloned PCR fragments from the bisulfite converted DNA. The bisulfite treated DNA was amplified by 18 PCR cycles and used for Solexa sequencing.

### Sequence Alignment and Identification of Methylcytosines

The reads generated by Illumina sequencing were aligned to the YH genome [11]. As DNA methylation has strand specificity, separate alignments of 6 Gbp in combined length were generated for the Watson and Crick strands of the YH genome. All cytosines in the 6 Gbp target sequence (“original form”) were replaced in silico by thymines (“alignment form”) to allow alignment after bisulfite conversion. In addition, the original forms of the reads were also transformed to cope with BS-treatment nucleotide conversion in the alignment process using the following criteria: (1) observed cytosines on the forward read of each read pair were in silico replaced by thymines, and (2) observed guanines on the reverse read of each read pair were in silico replaced by adenosines. We then mapped the “alignment form” reads to the “alignment form” target sequence using SOAPaligner [32]. Every hit with a single placement with minimum number of mismatches and a clear strand assignment was defined as an unambiguous alignment and was used in methylcytosine ascertainment. Ambiguously aligned reads were only used to estimate the approximate copy number of the local region. Local copy number of a genomic location was calculated by averaging the hit counts of all reads that cover a certain genomic location. Genomic bases with a copy number larger than 1.5 were not used to call methylcytosines and not used in any subsequent analysis to avoid errors caused by misalignment. In total, 2.21 Gbp (77.5% of the whole genome excluding N's) were of local copy number <1.5, which we defined as the “unique” part of genome that contained all cytosines analyzed in this study.

For methylcytosine identification, we transformed each aligned read and the two strands of the YH genome back to their original forms to build an alignment between the original forms. In the unique part of genome, cytosines that were covered by cytosines from reads on the same strand or guanines from those on the opposite strand (hereafter, referred to as ascertainment bases) were called as potentially methylated sites. To exclude spurious ascertainment bases that were caused by sequencing errors, we filtered out all bases with quality scores lower than 14. Increasing the quality threshold further did not change the non-CpG methylation rate. The false positive rate of methylcytosine identification was calculated as: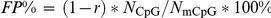 where *r* is the conversion rate (proportion of non-CpG cytosines with Q14 ascertainment bases), *N*_CpG_ is the total number of CpG cytosines, and *N*_mCpG_ is the total number of ascertained methylated CpG cytosines. As non-CpG methylation may occur, though at a very low level, the false positive rate is an overestimation.

### Estimation of Methylation Level

Sequencing errors could affect the ascertainment of methylation; therefore, we used the non-CpG methylation level as an indicator of errors. Overall methylation level of non-CpG sites becomes stable when quality >14 (Figure S9), which means the estimate is reliable above such a threshold. To eliminate the effect of low quality bases when estimating the methylation level of a specific genomic CpG cytosine, we divided the number of ascertainment bases by the number of total Q14-covering bases of that genomic location. To estimate the methylation level of a single base accurately, we only used CpG cytosines with a per-strand depth of more than 4 in the analysis of distribution of single CpG methylation levels, as for Figure 1 in the main text. Distribution of methylation level on CpG sites with 4× to 10× coverage, between which there was at minimum a consistent 10× coverage, indicated that the depth requirement was reasonable at even 4× coverage and could provide 5 different results (Figure S10).

For estimating the methylation level in a specific region, we divided the number of all ascertaining bases in the region by the number of all Q14 bases covering CpG cytosines in that region.

### Identification of Potential Tissue-Specific Differentially Methylated Regions (tDMR)

Putative tDMRs were identified by comparison of the PBMC and fetal lung fibroblast cell (IMR90) [2] methylomes using windows that contained at least 5 CpG sites with a 2-fold change in methylation level and Fisher test *p* value <1e−20. In addition, we require that both tissues should not be hypomethylated in tDMR discovery. Two nearby tDMRs would be considered interdependent and joined into one continuous tDMR if the genomic region from the start of an upstream tDMR to the end of a downstream tDMR also had 2-fold methylation level differences between sperm and PBMC with a *p* value <1e−20. Otherwise, the two tDMRs were viewed as independent. After iteratively merging interdependent tDMRs, the final dataset of tDMRs was made up of those that were independent from each other.

### Identification of Haploid Differentially Methylated Regions (hDMR)

We checked single-end and paired-end reads that were aligned across heterozygotes identified from the YH genome [11] to assign them to specific alleles. We calculated the methylation level of CpGs in SNP-allele containing reads that were assigned to an allele, and the number of methylcytosines and cytosines in the reads from each allele were subjected to Fisher test. Regions with at least 5 genomic CpGs, 2-fold change in methylation level, and a *p* value <0.001 were defined as hDMRs. Two hDMRs were joined if the phasing relationship could be validated by haplotype analysis of the corresponding YH sequence data or by reads spanning two heterozygotes.

## Supporting Information

Figure S1 **Cumulative distribution of sequencing depth in CpG sites.**
(0.73 MB EPS)

Figure S2 **Consistency in quality and methylation levels between randomly selected two libraries.**
(0.52 MB EPS)

Figure S3 **Distribution of methylation level of non-CpG cytosines with depth no less than 10×.**
(0.76 MB EPS)

Figure S4 **Methylation distribution of chromosomes 6, 20, and 22.**
(0.57 MB EPS)

Figure S5 **Methylation level of evolutionarily divergent Alu elements in human PBMC.**
(1.68 MB EPS)

Figure S6 **Co-methylation patterns for different genomic features.**
(2.54 MB EPS)

Figure S7 **Transformation of methylation correlation at nearby CpG cytosines.**
(1.80 MB EPS)

Figure S8 **Map of haploid differentially methylated regions (hDMR) and known imprinted genes in the local genomic spaces of imprinted clusters.**
(1.10 MB EPS)

Figure S9 **Inconversion rate (estimated by mean methylation level in non-CpG sites) at different quality score thresholds.**
(0.65 MB EPS)

Figure S10 **Methylation level distribution at CpG sites of 4–10-fold coverage and those of >10-fold coverage.**
(0.63 MB EPS)

Figure S11 **Canonical analysis of methylation level (red) and CpG density (blue) at CpG islands.**
(0.67 MB EPS)

Figure S12 **Co-methylation pattern of CpGs with local CpG density of 3%.**
(0.67 MB EPS)

Table S1 **Data production summary of PBMC methylome study.**
(0.11 MB PDF)

Table S2 **GO classification of PBMC-specific hypomethylated genes.**
(0.01 MB PDF)

Table S3 **GO classification of imr90-specific hypomethylated genes.**
(0.01 MB PDF)

Table S4 **Full list of hDMRs.**
(0.70 MB PDF)

Table S5 **Genes with hDMRs.**
(0.21 MB PDF)

Table S6 **Validation of expression level of both alleles for genes displaying allele-specific methylation.**
(0.01 MB PDF)

Table S7 **GO classification of allele-specific methylated genes.**
(0.06 MB PDF)

Table S8 **Validation of methylation level of CpG by Sanger sequencing.**
(0.03 MB PDF)

## Abbreviations

ASE: allele-specific expression
ASM: allele-specific methylation
BS-seq: bisulfite sequencing
hDMR: haploid-specific differentially methylated regions
PBMC: peripheral blood mononuclear cells
tDMR: tissue-specific differentially methylated regions
YH: YanHuang (the name of first Asian genome project)
