# Supplementary material for: The DNA Methylome of Human Peripheral Blood Mononuclear Cells
Source: PLoS Biol. 2010 Nov 9;8(11):e1000533. doi: 10.1371/journal.pbio.1000533 (PMC2976721; doi:10.1371/journal.pbio.1000533)
Supplement: Table S1 — Data production summary of PBMC methylome study. (0.11 MB PDF) [file pbio.1000533.s013.pdf]

Table S1. Data production summary of PBMC methylome study.

a)

| Library | Insert Size ± 3SD (bp) | Read Length (bp) | Reads Number | Raw Data (Gbp) |
|---|---|---|---|---|
| HUMtg1RABDCAAPE | 111±31 | 44 | 58,490,616 | 2.6 |
| | | 75 | 331,307,522 | 24.9 |
| HUMtg1RADSADPE | 182±46 | 44 | 45,119,798 | 2 |
| | | 75 | 366,998,694 | 27.3 |
| HUMtg2RABDCABPE | 151±43 | 44 | 41,469,338 | 1.8 |
| | | 75 | 252,028,298 | 18.9 |
| HUMtg2RABDCBAPE | 96±31 | 44 | 287,652,886 | 12.7 |
| | | 75 | - | - |
| HUMtg2RABDCBBPE | 135±37 | 44 | 60,369,744 | 2.7 |
| | | 75 | 144,023,246 | 10.8 |

b)

| Read length (bp) | # of raw reads | # of aligned Reads | | Aligned Bases (bp) | Effective strand depth | Unambiguous rate (%) | Mismatch rate (%) |
|---|---|---|---|---|---|---|---|
| 44 | 493,102,382 | SE | 159,881,472 | 6,779,415,037 | 1.2 | 87.85 | 1.1 |
| | | PE | 209,193,248 | 9,133,788,437 | 1.6 | 90.92 | 0.8 |
| 75 | 1,094,357,760 | SE | 398,503,878 | 24,941,188,529 | 4.3 | 82.69 | 1.8 |
| | | PE | 406,987,966 | 29,599,486,371 | 5.2 | 92.11 | 1.1 |
| Total | 1,587,460,142 | | 1,174,566,564 | 70,453,878,374 | 12.3 | 88.12 | 1.3 |
