# Supplementary material for: The DNA Methylome of Human Peripheral Blood Mononuclear Cells
Source: PLoS Biol. 2010 Nov 9;8(11):e1000533. doi: 10.1371/journal.pbio.1000533 (PMC2976721; doi:10.1371/journal.pbio.1000533)
Supplement: Table S2 — GO classification of PBMC-specific hypomethylated genes. (0.01 MB PDF) [file pbio.1000533.s014.pdf]

Table S2. GO classification of PBMC-specific hypomethylated genes.

GO category	*P* value	Over /under represent
DNA damage checkpoint	0.000491011	over
DNA-directed RNA polymerase II, core complex	0.001787826	over
nuclear mRNA splicing, via spliceosome	0.00316666	over
RNA splicing factor activity, transesterification mechanism	0.00344937	over
RNA splicing	0.003588529	over
intracellular	0.004397533	over
JAK-STAT cascade	0.004687771	over
DNA binding	0.004835307	over
blastocyst growth	0.004839991	over
phosphatidylinositol-3,4-bisphosphate 3-phosphatase activity	0.004839991	over
mitochondrial proton-transporting ATP synthase, catalytic core	0.004839991	over
regulation of protein amino acid phosphorylation	0.004839991	over
nuclear inclusion body	0.004839991	over
dipeptidyl-peptidase III activity	0.004839991	over
inositol phosphate dephosphorylation	0.004839991	over
sphinganine kinase activity	0.004839991	over
negative regulation of JAK-STAT cascade	0.004839991	over
phosphoinositide dephosphorylation	0.004839991	over
negative regulation of protein kinase B signaling cascade	0.004839991	over
sphinganine-1-phosphate biosynthetic process	0.004839991	over
inositol-1,3,4,5-tetrakisphosphate 3-phosphatase activity	0.004839991	over
negative regulation of cytokine biosynthetic process	0.004839991	over
ferritin complex	0.004839991	over
negative regulation of focal adhesion formation	0.004839991	over
DNA damage response, signal transduction by p53 class media	0.004839991	over
regulation of fibroblast proliferation	0.004839991	over
phosphatidylinositol-3-phosphatase activity	0.004839991	over
