# Supplementary material for: The DNA Methylome of Human Peripheral Blood Mononuclear Cells
Source: PLoS Biol. 2010 Nov 9;8(11):e1000533. doi: 10.1371/journal.pbio.1000533 (PMC2976721; doi:10.1371/journal.pbio.1000533)
Supplement: Table S3 — GO classification of imr90-specific hypomethylated genes. (0.01 MB PDF) [file pbio.1000533.s015.pdf]

Table S3. GO classification of imr90-specific hypomethylated genes.

GO category	*P* value	Over /under represent
nucleus	7.10E-07	under
transcription factor activity	0.000104627	under
sequence-specific DNA binding	0.000660161	under
serine-type endopeptidase activity	0.00080711	over
rhodopsin-like receptor activity	0.002032473	over
RNA binding	0.002686713	under
MHC class II receptor activity	0.00287155	under
symporter activity	0.003634038	over
cAMP binding	0.003682507	over
intracellular protein transport	0.003757302	under
ion transport	0.004213982	over
negative regulation of cell proliferation	0.004355368	under
