# Supplementary material for: The DNA Methylome of Human Peripheral Blood Mononuclear Cells
Source: PLoS Biol. 2010 Nov 9;8(11):e1000533. doi: 10.1371/journal.pbio.1000533 (PMC2976721; doi:10.1371/journal.pbio.1000533)
Supplement: Table S4 — Full list of hDMRs. (0.70 MB PDF) [file pbio.1000533.s016.pdf]

# Table S4. Full list of hDMRs.

| YH heterozygote | Chr | start position | end position | size(bp) | # of CpG | allele 1 methylation | | | allele 2 methylation | | | *P*-value (fisher test) | repeat level |
|---|---|---|---|---|---|---|---|---|---|---|---|---|---|
| | | | | | | allele | total | methylated | allele | total | methylated | | |
| chr10:1428597:M | chr10 | 1E+06 | 1E+06 | 253 | 13 | A | 14 | 13 | C | 14 | 2 | 3.48E-05 | 0 |
| chr10:1995396:S | chr10 | 2E+06 | 2E+06 | 408 | 10 | C | 17 | 17 | G | 6 | 1 | 0.00018 | 0 |
| chr10:2974699:W | chr10 | 3E+06 | 3E+06 | 378 | 7 | A | 32 | 22 | T | 31 | 6 | 0.00012 | 0 |
| chr10:6429511:R | chr10 | 6E+06 | 6E+06 | 323 | 9 | A | 15 | 7 | G | 37 | 1 | 0.00032 | 0 |
| chr10:6956611:M | chr10 | 7E+06 | 7E+06 | 322 | 6 | A | 11 | 3 | C | 30 | 26 | 0.0006 | 0 |
| chr10:11278201:R | chr10 | 1E+07 | 1E+07 | 191 | 5 | A | 4 | 0 | G | 29 | 27 | 0.00037 | 0 |
| chr10:46390045:R | chr10 | 5E+07 | 5E+07 | 290 | 17 | A | 54 | 44 | G | 24 | 9 | 0.00022 | 0 |
| chr10:49331990:S | chr10 | 5E+07 | 5E+07 | 350 | 8 | C | 8 | 2 | G | 20 | 20 | 7.43E-05 | 0 |
| chr10:97062308:M | chr10 | 1E+08 | 1E+08 | 263 | 5 | A | 14 | 3 | C | 27 | 23 | 0.0001 | 0 |
| chr10:102890248:S | chr10 | 1E+08 | 1E+08 | 186 | 19 | C | 22 | 9 | G | 43 | 1 | 0.00012 | 0 |
| chr10:115851453:K | chr10 | 1E+08 | 1E+08 | 236 | 8 | G | 10 | 0 | T | 18 | 12 | 0.00088 | 0 |
| chr10:123862117:Y | chr10 | 1E+08 | 1E+08 | 318 | 22 | C | 14 | 0 | T | 14 | 8 | 0.00097 | 0 |
| chr10:126346830:K | chr10 | 1E+08 | 1E+08 | 265 | 5 | G | 6 | 0 | T | 19 | 16 | 0.00047 | 0 |
| chr10:126671779:K | chr10 | 1E+08 | 1E+08 | 416 | 11 | G | 61 | 8 | T | 58 | 53 | 3.41E-19 | 0 |
| chr10:127574384:R | chr10 | 1E+08 | 1E+08 | 376 | 32 | A | 63 | 1 | G | 185 | 101 | 2.19E-16 | 0 |
| chr10:127574488:Y | chr10 | 1E+08 | 1E+08 | 173 | 17 | C | 40 | 6 | T | 53 | 37 | 1.07E-07 | 0 |
| chr10:127574499:R | chr10 | 1E+08 | 1E+08 | 343 | 37 | A | 87 | 59 | G | 82 | 8 | 1.68E-15 | 0 |
| chr10:127574788:S | chr10 | 1E+08 | 1E+08 | 376 | 39 | C | 202 | 126 | G | 25 | 0 | 2.48E-10 | 0 |
| chr10:127575080:M | chr10 | 1E+08 | 1E+08 | 388 | 46 | A | 277 | 208 | C | 176 | 61 | 1.89E-17 | 0 |
| chr10:127575425:K | chr10 | 1E+08 | 1E+08 | 400 | 29 | G | 428 | 269 | T | 97 | 4 | 2.59E-29 | 0 |
| chr10:127604847:K | chr10 | 1E+08 | 1E+08 | 322 | 5 | G | 65 | 21 | T | 32 | 27 | 1.23E-06 | 0 |
| chr10:130319319:S | chr10 | 1E+08 | 1E+08 | 319 | 5 | C | 14 | 9 | G | 12 | 0 | 0.00071 | 0 |
| chr10:132025695:S | chr10 | 1E+08 | 1E+08 | 258 | 11 | C | 8 | 1 | G | 30 | 25 | 0.00043 | 0 |
| chr10:133047289:W | chr10 | 1E+08 | 1E+08 | 338 | 6 | A | 12 | 1 | T | 11 | 10 | 0.00011 | 0 |
| chr10:135192305:S | chr10 | 1E+08 | 1E+08 | 264 | 23 | C | 57 | 1 | G | 11 | 8 | 1.92E-07 | 0 |
| chr11:179480:W | chr11 | 179375 | 179522 | 148 | 23 | A | 27 | 8 | T | 25 | 24 | 4.86E-07 | 0.0135 |
| chr11:487359:R | chr11 | 487286 | 487450 | 165 | 9 | A | 14 | 13 | G | 5 | 0 | 0.00052 | 0 |
| chr11:1007231:R | chr11 | 1E+06 | 1E+06 | 329 | 5 | A | 16 | 14 | G | 62 | 14 | 2.97E-06 | 0.0061 |
| chr11:1620616:Y | chr11 | 2E+06 | 2E+06 | 232 | 10 | C | 9 | 1 | T | 27 | 24 | 4.44E-05 | 0 |
| chr11:2126331:S | chr11 | 2E+06 | 2E+06 | 408 | 9 | C | 13 | 4 | G | 18 | 17 | 0.0003 | 0 |
| chr11:2787141:Y | chr11 | 3E+06 | 3E+06 | 258 | 8 | C | 9 | 9 | T | 6 | 0 | 0.0002 | 0 |
| chr11:11639624:R | chr11 | 1E+07 | 1E+07 | 204 | 7 | A | 20 | 19 | G | 4 | 0 | 0.00047 | 0 |
| chr11:19941915:S | chr11 | 2E+07 | 2E+07 | 395 | 9 | C | 15 | 8 | G | 20 | 0 | 0.00027 | 0 |
| chr11:43501001:M | chr11 | 4E+07 | 4E+07 | 254 | 8 | A | 24 | 21 | C | 98 | 5 | 5.61E-16 | 0.0236 |
| chr11:49830208:S | chr11 | 5E+07 | 5E+07 | 245 | 13 | C | 12 | 2 | G | 14 | 13 | 0.00017 | 0 |
| chr11:61264985:R | chr11 | 6E+07 | 6E+07 | 201 | 7 | A | 18 | 17 | G | 4 | 0 | 0.00068 | 0 |
| chr11:67174754:Y | chr11 | 7E+07 | 7E+07 | 229 | 10 | C | 5 | 5 | T | 21 | 0 | 1.52E-05 | 0 |
| chr11:113929139:Y | chr11 | 1E+08 | 1E+08 | 702 | 5 | C | 47 | 17 | T | 46 | 36 | 5.72E-05 | 0 |
| chr11:119910353:R | chr11 | 1E+08 | 1E+08 | 311 | 9 | A | 13 | 13 | G | 26 | 10 | 0.00028 | 0 |
| chr11:122184121:Y | chr11 | 1E+08 | 1E+08 | 208 | 5 | C | 7 | 0 | T | 11 | 10 | 0.00025 | 0 |
| chr11:133433475:Y | chr11 | 1E+08 | 1E+08 | 308 | 16 | C | 36 | 0 | T | 32 | 10 | 0.00022 | 0 |
| chr12:168079:Y | chr12 | 168029 | 168230 | 202 | 8 | C | 13 | 1 | T | 10 | 8 | 0.00073 | 0 |
| chr12:380722:Y | chr12 | 380578 | 380926 | 349 | 16 | C | 77 | 0 | T | 22 | 7 | 1.15E-05 | 0 |
| chr12:1330815:S | chr12 | 1E+06 | 1E+06 | 340 | 5 | C | 7 | 0 | G | 20 | 18 | 4.05E-05 | 0 |

| | | | | | | | | | | | | | |
|---|---|---|---|---|---|---|---|---|---|---|---|---|---|
| chr12:7672519:R | chr12 | 8E+06 | 8E+06 | 185 | 12 | A | 6 | 0 | G | 20 | 19 | 3.04E-05 | 0 |
| chr12:10155506:S | chr12 | 1E+07 | 1E+07 | 195 | 15 | C | 6 | 0 | G | 21 | 19 | 9.46E-05 | 0 |
| chr12:11062670:S | chr12 | 1E+07 | 1E+07 | 695 | 6 | C | 29 | 12 | G | 32 | 30 | 1.38E-05 | 0.0504 |
| chr12:11116497:M | chr12 | 1E+07 | 1E+07 | 407 | 7 | A | 22 | 19 | C | 5 | 0 | 0.00069 | 0 |
| chr12:31261695:Y | chr12 | 3E+07 | 3E+07 | 250 | 5 | C | 18 | 15 | T | 6 | 0 | 0.00062 | 0 |
| chr12:34170538:K | chr12 | 3E+07 | 3E+07 | 371 | 6 | G | 40 | 24 | T | 34 | 4 | 2.78E-05 | 0.0243 |
| chr12:34216617:M | chr12 | 3E+07 | 3E+07 | 492 | 5 | A | 44 | 33 | C | 153 | 53 | 2.27E-06 | 0.0833 |
| chr12:34261226:Y | chr12 | 3E+07 | 3E+07 | 348 | 8 | C | 33 | 9 | T | 48 | 43 | 9.89E-09 | 0.0747 |
| chr12:36779804:R | chr12 | 4E+07 | 4E+07 | 309 | 11 | A | 16 | 15 | G | 20 | 5 | 3.78E-05 | 0.0194 |
| chr12:40611140:K | chr12 | 4E+07 | 4E+07 | 367 | 5 | G | 22 | 4 | T | 23 | 16 | 0.00084 | 0 |
| chr12:50494909:S | chr12 | 5E+07 | 5E+07 | 369 | 28 | C | 14 | 12 | G | 20 | 1 | 1.98E-06 | 0 |
| chr12:50832665:R | chr12 | 5E+07 | 5E+07 | 307 | 17 | A | 19 | 1 | G | 14 | 13 | 3.26E-07 | 0 |
| chr12:51377833:M | chr12 | 5E+07 | 5E+07 | 236 | 7 | A | 19 | 19 | C | 3 | 0 | 0.00065 | 0 |
| chr12:56503583:S | chr12 | 6E+07 | 6E+07 | 294 | 8 | C | 14 | 13 | G | 52 | 0 | 6.85E-13 | 0 |
| chr12:56503965:K | chr12 | 6E+07 | 6E+07 | 292 | 11 | G | 42 | 34 | T | 40 | 13 | 1.50E-05 | 0 |
| chr12:56509545:R | chr12 | 6E+07 | 6E+07 | 340 | 6 | A | 44 | 30 | G | 16 | 1 | 1.85E-05 | 0 |
| chr12:74242121:Y | chr12 | 7E+07 | 7E+07 | 388 | 7 | C | 18 | 1 | T | 11 | 9 | 5.00E-05 | 0 |
| chr12:110944530:K | chr12 | 1E+08 | 1E+08 | 305 | 7 | G | 17 | 5 | T | 13 | 13 | 8.70E-05 | 0 |
| chr12:120099514:S | chr12 | 1E+08 | 1E+08 | 307 | 12 | C | 32 | 11 | G | 47 | 40 | 8.14E-06 | 0 |
| chr12:120840540:Y | chr12 | 1E+08 | 1E+08 | 288 | 12 | C | 13 | 12 | T | 10 | 1 | 0.00011 | 0 |
| chr12:123602292:R | chr12 | 1E+08 | 1E+08 | 209 | 7 | A | 11 | 0 | G | 12 | 9 | 0.00034 | 0 |
| chr12:123787670:R | chr12 | 1E+08 | 1E+08 | 305 | 6 | A | 15 | 13 | G | 6 | 0 | 0.00052 | 0 |
| chr12:128041205:Y | chr12 | 1E+08 | 1E+08 | 250 | 5 | C | 22 | 21 | T | 9 | 3 | 0.00072 | 0 |
| chr12:130743527:K | chr12 | 1E+08 | 1E+08 | 171 | 5 | G | 8 | 8 | T | 9 | 0 | 4.11E-05 | 0 |
| chr12:130902574:Y | chr12 | 1E+08 | 1E+08 | 230 | 13 | C | 8 | 1 | T | 22 | 21 | 3.02E-05 | 0 |
| chr12:131103205:R | chr12 | 1E+08 | 1E+08 | 183 | 10 | A | 20 | 18 | G | 10 | 0 | 2.20E-06 | 0 |
| chr12:131253028:K | chr12 | 1E+08 | 1E+08 | 270 | 5 | G | 14 | 7 | T | 22 | 22 | 0.00041 | 0 |
| chr13:18541183:Y | chr13 | 2E+07 | 2E+07 | 441 | 7 | C | 40 | 27 | T | 10 | 0 | 0.00011 | 0.0975 |
| chr13:23375498:R | chr13 | 2E+07 | 2E+07 | 207 | 21 | A | 38 | 2 | G | 20 | 9 | 0.00055 | 0 |
| chr13:26382992:R | chr13 | 3E+07 | 3E+07 | 391 | 5 | A | 25 | 22 | G | 5 | 0 | 0.00039 | 0 |
| chr13:48793645:R | chr13 | 5E+07 | 5E+07 | 280 | 10 | A | 16 | 8 | G | 22 | 22 | 0.00026 | 0 |
| chr13:49294657:W | chr13 | 5E+07 | 5E+07 | 377 | 5 | A | 24 | 12 | T | 19 | 19 | 0.0003 | 0.0557 |
| chr13:62543854:R | chr13 | 6E+07 | 6E+07 | 1468 | 7 | A | 77 | 8 | G | 91 | 54 | 1.60E-11 | 0.0007 |
| chr13:97994122:M | chr13 | 1E+08 | 1E+08 | 212 | 10 | A | 17 | 10 | C | 17 | 0 | 0.00015 | 0 |
| chr13:99433607:R | chr13 | 1E+08 | 1E+08 | 314 | 14 | A | 29 | 0 | G | 21 | 8 | 0.00038 | 0 |
| chr13:99954646:R | chr13 | 1E+08 | 1E+08 | 212 | 9 | A | 6 | 1 | G | 32 | 31 | 6.99E-05 | 0 |
| chr13:100866569:K | chr13 | 1E+08 | 1E+08 | 102 | 15 | G | 14 | 11 | T | 23 | 1 | 4.57E-06 | 0 |
| chr13:107144419:K | chr13 | 1E+08 | 1E+08 | 434 | 7 | G | 16 | 7 | T | 30 | 28 | 0.00039 | 0 |
| chr13:110069271:R | chr13 | 1E+08 | 1E+08 | 244 | 6 | A | 5 | 0 | G | 14 | 13 | 0.00052 | 0 |
| chr13:113607931:S | chr13 | 1E+08 | 1E+08 | 286 | 22 | C | 21 | 19 | G | 28 | 11 | 0.00032 | 0 |
| chr13:113909298:Y | chr13 | 1E+08 | 1E+08 | 295 | 14 | C | 4 | 0 | T | 18 | 17 | 0.00068 | 0.0475 |
| chr13:114065598:S | chr13 | 1E+08 | 1E+08 | 188 | 21 | C | 39 | 25 | G | 71 | 8 | 1.36E-08 | 0.0798 |
| chr14:22486303:M | chr14 | 2E+07 | 2E+07 | 405 | 6 | A | 33 | 27 | C | 5 | 0 | 0.00092 | 0 |
| chr14:23711366:Y | chr14 | 2E+07 | 2E+07 | 239 | 10 | C | 11 | 10 | T | 11 | 1 | 0.00017 | 0 |
| chr14:32023501:W | chr14 | 3E+07 | 3E+07 | 468 | 15 | T | 28 | 4 | A | 998 | 9 | 0.00027 | 0.0085 |
| chr14:34254292:Y | chr14 | 3E+07 | 3E+07 | 315 | 5 | C | 10 | 2 | T | 12 | 12 | 0.00014 | 0 |
| chr14:53435871:Y | chr14 | 5E+07 | 5E+07 | 435 | 7 | C | 14 | 14 | T | 23 | 11 | 0.00087 | 0 |
| chr14:54991314:S | chr14 | 5E+07 | 5E+07 | 312 | 7 | C | 40 | 17 | G | 24 | 21 | 0.0005 | 0 |
| chr14:55541458:K | chr14 | 6E+07 | 6E+07 | 318 | 9 | G | 38 | 0 | T | 16 | 11 | 4.56E-08 | 0 |
| chr14:64329980:K | chr14 | 6E+07 | 6E+07 | 202 | 8 | G | 15 | 13 | T | 11 | 1 | 0.00017 | 0 |
| chr14:65474186:Y | chr14 | 7E+07 | 7E+07 | 357 | 5 | C | 19 | 17 | T | 22 | 4 | 5.75E-06 | 0 |

| | | | | | | | | | | | | | |
|---|---|---|---|---|---|---|---|---|---|---|---|---|---|
| chr14:76560422:M | chr14 | 8E+07 | 8E+07 | 313 | 12 | A | 36 | 2 | C | 16 | 10 | 2.52E-05 | 0 |
| chr14:77449437:Y | chr14 | 8E+07 | 8E+07 | 272 | 5 | C | 6 | 6 | T | 8 | 0 | 0.00033 | 0 |
| chr14:78780542:Y | chr14 | 8E+07 | 8E+07 | 281 | 5 | C | 10 | 2 | T | 19 | 17 | 0.00039 | 0 |
| chr14:100363281:M | chr14 | 1E+08 | 1E+08 | 202 | 11 | A | 23 | 16 | C | 12 | 0 | 6.26E-05 | 0 |
| chr14:100572054:Y | chr14 | 1E+08 | 1E+08 | 199 | 5 | C | 12 | 11 | T | 6 | 0 | 0.00038 | 0 |
| chr14:102851852:S | chr14 | 1E+08 | 1E+08 | 323 | 7 | C | 20 | 17 | G | 21 | 6 | 0.00043 | 0 |
| chr14:103646201:R | chr14 | 1E+08 | 1E+08 | 193 | 6 | A | 10 | 9 | G | 8 | 0 | 0.00021 | 0 |
| chr14:104607207:Y | chr14 | 1E+08 | 1E+08 | 265 | 9 | C | 34 | 30 | T | 7 | 0 | 1.47E-05 | 0 |
| chr14:104779371:R | chr14 | 1E+08 | 1E+08 | 241 | 7 | A | 15 | 14 | G | 6 | 0 | 0.00013 | 0 |
| chr14:105752519:S | chr14 | 1E+08 | 1E+08 | 232 | 5 | C | 9 | 0 | G | 8 | 7 | 0.00041 | 0 |
| chr15:18760480:S | chr15 | 2E+07 | 2E+07 | 194 | 26 | C | 18 | 14 | G | 18 | 0 | 8.06E-07 | 0.0515 |
| chr15:19221433:S | chr15 | 2E+07 | 2E+07 | 320 | 32 | C | 118 | 30 | G | 44 | 25 | 0.00032 | 0 |
| chr15:19221635:S | chr15 | 2E+07 | 2E+07 | 339 | 19 | C | 89 | 32 | G | 76 | 56 | 2.03E-06 | 0.0885 |
| chr15:19241223:M | chr15 | 2E+07 | 2E+07 | 327 | 40 | A | 67 | 52 | C | 48 | 7 | 9.67E-12 | 0 |
| chr15:21665778:K | chr15 | 2E+07 | 2E+07 | 250 | 17 | G | 32 | 1 | T | 7 | 6 | 1.46E-05 | 0 |
| chr15:23907821:S | chr15 | 2E+07 | 2E+07 | 241 | 7 | C | 21 | 19 | G | 21 | 6 | 4.82E-05 | 0 |
| chr15:24518540:S | chr15 | 2E+07 | 2E+07 | 307 | 5 | C | 17 | 5 | G | 27 | 23 | 0.00031 | 0 |
| chr15:27183361:Y | chr15 | 3E+07 | 3E+07 | 237 | 20 | C | 19 | 13 | T | 12 | 0 | 0.00014 | 0 |
| chr15:29587002:R | chr15 | 3E+07 | 3E+07 | 193 | 5 | A | 14 | 13 | G | 10 | 0 | 5.61E-06 | 0 |
| chr15:32658265:M | chr15 | 3E+07 | 3E+07 | 259 | 5 | A | 13 | 2 | C | 65 | 52 | 1.69E-05 | 0 |
| chr15:49700263:R | chr15 | 5E+07 | 5E+07 | 373 | 5 | A | 12 | 2 | G | 14 | 14 | 1.24E-05 | 0 |
| chr15:76910034:R | chr15 | 8E+07 | 8E+07 | 236 | 14 | A | 7 | 1 | G | 20 | 19 | 0.00016 | 0 |
| chr15:82348680:K | chr15 | 8E+07 | 8E+07 | 329 | 5 | G | 10 | 10 | T | 18 | 6 | 0.00088 | 0 |
| chr15:83752652:R | chr15 | 8E+07 | 8E+07 | 333 | 6 | A | 22 | 15 | G | 42 | 9 | 0.00038 | 0 |
| chr15:84112597:M | chr15 | 8E+07 | 8E+07 | 193 | 10 | A | 5 | 0 | C | 30 | 28 | 6.47E-05 | 0 |
| chr15:87053182:S | chr15 | 9E+07 | 9E+07 | 318 | 7 | C | 14 | 11 | G | 9 | 0 | 0.00034 | 0 |
| chr15:87761290:W | chr15 | 9E+07 | 9E+07 | 217 | 10 | A | 17 | 0 | T | 15 | 8 | 0.00061 | 0.0184 |
| chr15:88546567:R | chr15 | 9E+07 | 9E+07 | 180 | 8 | A | 9 | 0 | G | 19 | 13 | 0.00083 | 0 |
| chr15:97467811:S | chr15 | 1E+08 | 1E+08 | 319 | 6 | C | 20 | 15 | G | 18 | 2 | 9.10E-05 | 0 |
| chr15:99462251:R | chr15 | 1E+08 | 1E+08 | 253 | 7 | A | 6 | 0 | G | 14 | 13 | 0.00018 | 0 |
| chr16:534472:M | chr16 | 534414 | 534582 | 169 | 11 | A | 26 | 25 | C | 18 | 6 | 9.46E-06 | 0 |
| chr16:798095:R | chr16 | 798032 | 798199 | 168 | 11 | A | 37 | 36 | G | 13 | 0 | 3.95E-11 | 0 |
| chr16:920089:R | chr16 | 919902 | 920251 | 350 | 15 | A | 21 | 21 | G | 8 | 2 | 5.89E-05 | 0 |
| chr16:3490148:M | chr16 | 3E+06 | 3E+06 | 214 | 18 | A | 34 | 2 | C | 12 | 7 | 0.00042 | 0 |
| chr16:4081729:Y | chr16 | 4E+06 | 4E+06 | 219 | 6 | C | 12 | 12 | T | 9 | 0 | 3.40E-06 | 0.0685 |
| chr16:6815929:Y | chr16 | 7E+06 | 7E+06 | 225 | 8 | C | 9 | 2 | T | 24 | 21 | 0.00081 | 0 |
| chr16:10112679:W | chr16 | 1E+07 | 1E+07 | 244 | 9 | A | 13 | 4 | T | 16 | 15 | 0.00099 | 0 |
| chr16:14301774:M | chr16 | 1E+07 | 1E+07 | 280 | 6 | A | 23 | 22 | C | 17 | 8 | 0.00068 | 0.0143 |
| chr16:28525472:Y | chr16 | 3E+07 | 3E+07 | 265 | 5 | C | 11 | 1 | T | 17 | 15 | 4.97E-05 | 0 |
| chr16:31109809:R | chr16 | 3E+07 | 3E+07 | 329 | 8 | A | 7 | 0 | G | 20 | 20 | 1.13E-06 | 0 |
| chr16:32393758:Y | chr16 | 3E+07 | 3E+07 | 295 | 11 | C | 34 | 11 | T | 56 | 43 | 4.88E-05 | 0 |
| chr16:33377968:R | chr16 | 3E+07 | 3E+07 | 284 | 5 | A | 20 | 17 | G | 97 | 40 | 0.00041 | 0.0176 |
| chr16:33447540:S | chr16 | 3E+07 | 3E+07 | 728 | 5 | C | 57 | 16 | G | 27 | 21 | 3.85E-05 | 0.0824 |
| chr16:33833070:R | chr16 | 3E+07 | 3E+07 | 405 | 5 | A | 82 | 20 | G | 66 | 43 | 8.76E-07 | 0.0765 |
| chr16:33868978:Y | chr16 | 3E+07 | 3E+07 | 129 | 19 | C | 370 | 105 | T | 34 | 22 | 5.61E-05 | 0 |
| chr16:33869683:Y | chr16 | 3E+07 | 3E+07 | 197 | 20 | C | 115 | 24 | T | 35 | 29 | 4.30E-11 | 0 |
| chr16:33871962:Y | chr16 | 3E+07 | 3E+07 | 133 | 16 | C | 37 | 13 | T | 8 | 8 | 0.00094 | 0 |
| chr16:46589175:W | chr16 | 5E+07 | 5E+07 | 346 | 6 | A | 41 | 37 | T | 22 | 8 | 1.32E-05 | 0 |
| chr16:69470887:R | chr16 | 7E+07 | 7E+07 | 238 | 6 | A | 16 | 15 | G | 20 | 6 | 0.00013 | 0 |
| chr16:69512693:R | chr16 | 7E+07 | 7E+07 | 214 | 6 | A | 15 | 6 | G | 24 | 22 | 0.00087 | 0 |
| chr16:69619885:Y | chr16 | 7E+07 | 7E+07 | 415 | 5 | C | 38 | 30 | T | 44 | 8 | 3.86E-08 | 0 |

| | | | | | | | | | | | | | |
|---|---|---|---|---|---|---|---|---|---|---|---|---|---|
| chr16:71917802:K | chr16 | 7E+07 | 7E+07 | 292 | 7 | G | 16 | 15 | T | 10 | 2 | 0.00023 | 0 |
| chr16:73434113:R | chr16 | 7E+07 | 7E+07 | 216 | 6 | A | 13 | 0 | G | 9 | 9 | 2.01E-06 | 0 |
| chr16:78009255:Y | chr16 | 8E+07 | 8E+07 | 236 | 10 | C | 23 | 21 | T | 10 | 0 | 7.13E-07 | 0 |
| chr16:79848365:Y | chr16 | 8E+07 | 8E+07 | 293 | 5 | C | 6 | 1 | T | 27 | 25 | 0.0005 | 0.041 |
| chr16:83859489:Y | chr16 | 8E+07 | 8E+07 | 150 | 5 | C | 13 | 13 | T | 12 | 3 | 0.00011 | 0 |
| chr16:87097703:M | chr16 | 9E+07 | 9E+07 | 212 | 6 | A | 18 | 14 | C | 17 | 2 | 0.00013 | 0 |
| chr16:87296901:R | chr16 | 9E+07 | 9E+07 | 243 | 14 | A | 25 | 14 | G | 26 | 0 | 3.45E-06 | 0 |
| chr16:87527804:K | chr16 | 9E+07 | 9E+07 | 260 | 10 | G | 9 | 4 | T | 24 | 24 | 0.00053 | 0.0308 |
| chr16:87674904:R | chr16 | 9E+07 | 9E+07 | 235 | 7 | A | 31 | 31 | G | 4 | 1 | 0.00061 | 0 |
| chr16:88268110:Y | chr16 | 9E+07 | 9E+07 | 110 | 7 | C | 12 | 12 | T | 5 | 0 | 0.00016 | 0 |
| chr17:30159:R | chr17 | 29998 | 30315 | 318 | 26 | A | 27 | 0 | G | 15 | 6 | 0.00095 | 0 |
| chr17:84936:R | chr17 | 84768 | 85007 | 240 | 12 | A | 8 | 0 | G | 22 | 20 | 7.69E-06 | 0 |
| chr17:1477461:M | chr17 | 1E+06 | 1E+06 | 215 | 6 | A | 26 | 1 | C | 12 | 9 | 1.22E-05 | 0 |
| chr17:5086283:Y | chr17 | 5E+06 | 5E+06 | 306 | 7 | C | 20 | 0 | T | 17 | 10 | 5.58E-05 | 0 |
| chr17:6499053:R | chr17 | 6E+06 | 6E+06 | 121 | 11 | A | 8 | 5 | G | 22 | 0 | 0.00039 | 0 |
| chr17:8943190:R | chr17 | 9E+06 | 9E+06 | 245 | 6 | A | 14 | 14 | G | 9 | 2 | 0.00015 | 0.0041 |
| chr17:17345221:Y | chr17 | 2E+07 | 2E+07 | 355 | 6 | C | 12 | 10 | T | 7 | 0 | 0.00071 | 0 |
| chr17:17345249:R | chr17 | 2E+07 | 2E+07 | 333 | 10 | A | 9 | 5 | G | 23 | 0 | 0.00063 | 0 |
| chr17:20723886:S | chr17 | 2E+07 | 2E+07 | 368 | 6 | C | 47 | 38 | G | 24 | 6 | 7.05E-06 | 0 |
| chr17:21154730:Y | chr17 | 2E+07 | 2E+07 | 363 | 10 | C | 9 | 0 | T | 38 | 24 | 0.0006 | 0 |
| chr17:21160894:S | chr17 | 2E+07 | 2E+07 | 318 | 25 | C | 93 | 31 | G | 70 | 5 | 4.68E-05 | 0 |
| chr17:21166983:Y | chr17 | 2E+07 | 2E+07 | 321 | 16 | C | 13 | 6 | T | 50 | 0 | 2.53E-05 | 0.0436 |
| chr17:21270012:K | chr17 | 2E+07 | 2E+07 | 491 | 7 | T | 27 | 8 | G | 36 | 27 | 0.00071 | 0.0896 |
| chr17:21467760:W | chr17 | 2E+07 | 2E+07 | 387 | 5 | A | 51 | 9 | T | 41 | 29 | 4.72E-07 | 0 |
| chr17:21749692:K | chr17 | 2E+07 | 2E+07 | 238 | 11 | G | 33 | 31 | T | 36 | 15 | 2.74E-06 | 0 |
| chr17:22336197:R | chr17 | 2E+07 | 2E+07 | 340 | 7 | A | 40 | 14 | G | 33 | 27 | 0.00011 | 0 |
| chr17:38793439:Y | chr17 | 4E+07 | 4E+07 | 240 | 10 | C | 30 | 2 | T | 12 | 8 | 0.00015 | 0 |
| chr17:38794088:Y | chr17 | 4E+07 | 4E+07 | 469 | 32 | C | 75 | 0 | T | 87 | 25 | 4.18E-08 | 0 |
| chr17:38819292:S | chr17 | 4E+07 | 4E+07 | 319 | 14 | C | 971 | 830 | G | 42 | 17 | 9.44E-11 | 0 |
| chr17:38822018:Y | chr17 | 4E+07 | 4E+07 | 400 | 26 | C | 706 | 480 | T | 44 | 1 | 2.11E-19 | 0 |
| chr17:38822148:S | chr17 | 4E+07 | 4E+07 | 346 | 21 | C | 103 | 58 | G | 39 | 1 | 4.03E-10 | 0 |
| chr17:38822325:Y | chr17 | 4E+07 | 4E+07 | 238 | 6 | C | 266 | 142 | T | 12 | 0 | 0.00015 | 0 |
| chr17:42621661:S | chr17 | 4E+07 | 4E+07 | 312 | 28 | C | ### | 1131 | G | 58 | 10 | 1.85E-29 | 0.0641 |
| chr17:45425740:R | chr17 | 5E+07 | 5E+07 | 189 | 20 | A | 8 | 6 | G | 29 | 0 | 1.20E-05 | 0 |
| chr17:58044277:M | chr17 | 6E+07 | 6E+07 | 357 | 5 | A | 33 | 30 | C | 13 | 3 | 1.57E-05 | 0 |
| chr17:61707567:S | chr17 | 6E+07 | 6E+07 | 334 | 5 | C | 18 | 16 | G | 14 | 4 | 0.00079 | 0 |
| chr17:63695187:R | chr17 | 6E+07 | 6E+07 | 484 | 6 | A | 46 | 40 | G | 17 | 6 | 0.00012 | 0.0744 |
| chr17:71529149:K | chr17 | 7E+07 | 7E+07 | 205 | 10 | G | 16 | 15 | T | 5 | 0 | 0.00029 | 0 |
| chr17:73982961:R | chr17 | 7E+07 | 7E+07 | 168 | 6 | A | 12 | 12 | G | 6 | 1 | 0.0007 | 0 |
| chr17:73983274:R | chr17 | 7E+07 | 7E+07 | 180 | 8 | A | 9 | 1 | G | 20 | 16 | 0.00086 | 0 |
| chr17:74710619:M | chr17 | 7E+07 | 7E+07 | 333 | 6 | A | 19 | 13 | C | 14 | 0 | 5.04E-05 | 0.015 |
| chr17:75660805:R | chr17 | 8E+07 | 8E+07 | 293 | 5 | A | 8 | 1 | G | 9 | 9 | 0.00041 | 0 |
| chr17:76577527:R | chr17 | 8E+07 | 8E+07 | 307 | 15 | A | 22 | 6 | G | 42 | 0 | 0.001 | 0 |
| chr17:77041649:S | chr17 | 8E+07 | 8E+07 | 181 | 7 | C | 9 | 8 | G | 22 | 4 | 0.00048 | 0 |
| chr18:12602345:Y | chr18 | 1E+07 | 1E+07 | 444 | 6 | C | 30 | 28 | T | 14 | 4 | 2.12E-05 | 0 |
| chr18:14421379:R | chr18 | 1E+07 | 1E+07 | 240 | 27 | A | 35 | 0 | G | 32 | 19 | 1.44E-08 | 0 |
| chr18:42590883:M | chr18 | 4E+07 | 4E+07 | 320 | 33 | A | 23 | 0 | C | 19 | 8 | 0.00064 | 0 |
| chr18:45538343:R | chr18 | 5E+07 | 5E+07 | 221 | 6 | A | 10 | 10 | G | 7 | 1 | 0.00057 | 0 |
| chr18:45974364:K | chr18 | 5E+07 | 5E+07 | 250 | 14 | G | 47 | 0 | T | 32 | 8 | 0.0004 | 0 |
| chr18:46901871:Y | chr18 | 5E+07 | 5E+07 | 91 | 5 | C | 5 | 0 | T | 8 | 8 | 0.00078 | 0 |
| chr18:50016426:W | chr18 | 5E+07 | 5E+07 | 308 | 8 | A | 41 | 39 | T | 15 | 6 | 2.84E-05 | 0.0649 |

| | | | | | | | | | | | | | |
|---|---|---|---|---|---|---|---|---|---|---|---|---|---|
| chr18:53620484:K | chr18 | 5E+07 | 5E+07 | 284 | 15 | G | 11 | 7 | T | 29 | 0 | 1.77E-05 | 0.0951 |
| chr18:56480961:R | chr18 | 6E+07 | 6E+07 | 525 | 19 | A | 113 | 41 | G | 94 | 70 | 3.82E-08 | 0.0114 |
| chr18:72245281:R | chr18 | 7E+07 | 7E+07 | 305 | 8 | A | 18 | 16 | G | 8 | 1 | 0.0004 | 0 |
| chr18:72947624:Y | chr18 | 7E+07 | 7E+07 | 361 | 5 | C | 16 | 5 | T | 10 | 10 | 0.00072 | 0 |
| chr18:75373624:Y | chr18 | 8E+07 | 8E+07 | 163 | 10 | C | 13 | 13 | T | 5 | 0 | 0.00012 | 0 |
| chr18:75774550:R | chr18 | 8E+07 | 8E+07 | 115 | 9 | A | 8 | 0 | G | 5 | 5 | 0.00078 | 0 |
| chr19:1803078:S | chr19 | 2E+06 | 2E+06 | 123 | 14 | C | 22 | 0 | G | 18 | 15 | 2.03E-08 | 0 |
| chr19:3320572:K | chr19 | 3E+06 | 3E+06 | 254 | 14 | G | 33 | 2 | T | 25 | 11 | 0.00098 | 0.0276 |
| chr19:12737964:S | chr19 | 1E+07 | 1E+07 | 193 | 11 | C | 20 | 17 | G | 17 | 0 | 9.31E-08 | 0 |
| chr19:15972787:Y | chr19 | 2E+07 | 2E+07 | 515 | 9 | C | 92 | 83 | T | 47 | 10 | 3.06E-16 | 0.0621 |
| chr19:17458830:K | chr19 | 2E+07 | 2E+07 | 156 | 9 | G | 14 | 14 | T | 5 | 0 | 8.60E-05 | 0 |
| chr19:18118750:Y | chr19 | 2E+07 | 2E+07 | 170 | 13 | C | 12 | 0 | T | 8 | 8 | 7.94E-06 | 0 |
| chr19:21465098:Y | chr19 | 2E+07 | 2E+07 | 292 | 5 | C | 17 | 17 | T | 3 | 0 | 0.00088 | 0 |
| chr19:23977338:R | chr19 | 2E+07 | 2E+07 | 196 | 11 | A | 24 | 18 | G | 700 | 193 | 3.00E-06 | 0 |
| chr19:33023189:Y | chr19 | 3E+07 | 3E+07 | 499 | 9 | C | 44 | 32 | T | 37 | 8 | 5.76E-06 | 0.0381 |
| chr19:37875509:M | chr19 | 4E+07 | 4E+07 | 163 | 7 | A | 22 | 0 | C | 15 | 7 | 0.00063 | 0 |
| chr19:41335535:K | chr19 | 4E+07 | 4E+07 | 256 | 9 | G | 18 | 4 | T | 26 | 21 | 0.00017 | 0 |
| chr19:44157852:W | chr19 | 4E+07 | 4E+07 | 191 | 13 | A | 15 | 9 | T | 20 | 1 | 0.00056 | 0 |
| chr19:49345887:M | chr19 | 5E+07 | 5E+07 | 311 | 5 | A | 28 | 0 | C | 16 | 14 | 1.04E-09 | 0.0257 |
| chr19:50182410:R | chr19 | 5E+07 | 5E+07 | 158 | 10 | A | 10 | 10 | G | 4 | 0 | 0.001 | 0 |
| chr19:55104029:S | chr19 | 6E+07 | 6E+07 | 99 | 5 | C | 8 | 8 | G | 7 | 0 | 0.00016 | 0 |
| chr19:56658743:R | chr19 | 6E+07 | 6E+07 | 333 | 14 | A | 18 | 8 | G | 28 | 0 | 0.00017 | 0.0901 |
| chr19:58970674:K | chr19 | 6E+07 | 6E+07 | 338 | 14 | G | 21 | 10 | T | 16 | 16 | 0.00061 | 0 |
| chr19:62042275:Y | chr19 | 6E+07 | 6E+07 | 342 | 17 | C | 11 | 2 | T | 20 | 17 | 0.00046 | 0 |
| chr19:62043863:Y | chr19 | 6E+07 | 6E+07 | 201 | 13 | C | 7 | 6 | T | 11 | 0 | 0.00038 | 0 |
| chr19:63247744:S | chr19 | 6E+07 | 6E+07 | 309 | 28 | C | 35 | 28 | G | 29 | 6 | 3.19E-06 | 0 |
| chr19:63559937:K | chr19 | 6E+07 | 6E+07 | 294 | 31 | G | 15 | 13 | T | 81 | 6 | 6.14E-10 | 0 |
| chr1:3133631:Y | chr1 | 3E+06 | 3E+06 | 229 | 12 | C | 10 | 2 | T | 12 | 12 | 0.00014 | 0 |
| chr1:3336208:S | chr1 | 3E+06 | 3E+06 | 198 | 7 | C | 26 | 20 | G | 11 | 1 | 0.0002 | 0 |
| chr1:5990158:Y | chr1 | 6E+06 | 6E+06 | 304 | 14 | C | 10 | 10 | T | 57 | 10 | 7.45E-07 | 0 |
| chr1:7425882:M | chr1 | 7E+06 | 7E+06 | 251 | 6 | A | 21 | 5 | C | 17 | 17 | 1.04E-06 | 0 |
| chr1:10633865:Y | chr1 | 1E+07 | 1E+07 | 141 | 10 | C | 4 | 0 | T | 25 | 25 | 4.21E-05 | 0 |
| chr1:15145768:K | chr1 | 2E+07 | 2E+07 | 222 | 7 | G | 5 | 1 | T | 18 | 18 | 0.00056 | 0.0495 |
| chr1:16264378:Y | chr1 | 2E+07 | 2E+07 | 340 | 7 | C | 10 | 1 | T | 17 | 14 | 0.00075 | 0 |
| chr1:16731408:K | chr1 | 2E+07 | 2E+07 | 368 | 10 | G | 12 | 0 | T | 31 | 24 | 3.29E-06 | 0 |
| chr1:16733829:W | chr1 | 2E+07 | 2E+07 | 278 | 21 | A | 67 | 32 | T | 34 | 4 | 0.00036 | 0 |
| chr1:16734149:M | chr1 | 2E+07 | 2E+07 | 367 | 25 | A | 59 | 1 | C | 148 | 50 | 7.17E-08 | 0 |
| chr1:16734728:S | chr1 | 2E+07 | 2E+07 | 301 | 30 | C | 26 | 3 | G | 90 | 69 | 2.20E-09 | 0 |
| chr1:16847345:R | chr1 | 2E+07 | 2E+07 | 359 | 29 | A | 86 | 37 | G | 48 | 7 | 0.00097 | 0 |
| chr1:16857487:R | chr1 | 2E+07 | 2E+07 | 264 | 23 | A | 68 | 48 | G | 34 | 7 | 2.13E-06 | 0 |
| chr1:16891185:R | chr1 | 2E+07 | 2E+07 | 285 | 12 | A | 16 | 3 | G | 27 | 25 | 1.32E-06 | 0 |
| chr1:16895697:R | chr1 | 2E+07 | 2E+07 | 327 | 22 | A | 4 | 4 | G | 70 | 3 | 3.04E-05 | 0.0153 |
| chr1:17071953:S | chr1 | 2E+07 | 2E+07 | 308 | 20 | C | 66 | 33 | G | 123 | 14 | 1.23E-08 | 0 |
| chr1:17072202:S | chr1 | 2E+07 | 2E+07 | 394 | 27 | C | 160 | 56 | G | 196 | 14 | 3.23E-11 | 0 |
| chr1:17072586:S | chr1 | 2E+07 | 2E+07 | 305 | 22 | C | 11 | 10 | G | 231 | 74 | 0.00014 | 0 |
| chr1:18551953:Y | chr1 | 2E+07 | 2E+07 | 205 | 8 | C | 6 | 0 | T | 15 | 13 | 0.00052 | 0 |
| chr1:20422959:Y | chr1 | 2E+07 | 2E+07 | 325 | 12 | C | 38 | 26 | T | 32 | 7 | 0.00012 | 0 |
| chr1:21635719:W | chr1 | 2E+07 | 2E+07 | 321 | 23 | A | 25 | 19 | T | 18 | 2 | 3.46E-05 | 0 |
| chr1:21737466:Y | chr1 | 2E+07 | 2E+07 | 250 | 16 | C | 20 | 2 | T | 30 | 22 | 1.04E-05 | 0 |
| chr1:22583138:S | chr1 | 2E+07 | 2E+07 | 354 | 10 | C | 22 | 9 | G | 20 | 19 | 0.00023 | 0 |
| chr1:28295434:Y | chr1 | 3E+07 | 3E+07 | 317 | 11 | C | 17 | 17 | T | 10 | 0 | 1.19E-07 | 0 |

| | | | | | | | | | | | | | | |
|---|---|---|---|---|---|---|---|---|---|---|---|---|---|---|
| chr1:29801024:Y | chr1 | 3E+07 | 3E+07 | 325 | 7 | C | 23 | 18 | T | 15 | 3 | 0.0007 | 0.0954 |
| chr1:32040924:M | chr1 | 3E+07 | 3E+07 | 333 | 6 | A | 24 | 0 | C | 32 | 16 | 1.55E-05 | 0 |
| chr1:77209519:S | chr1 | 8E+07 | 8E+07 | 445 | 8 | C | 674 | 6 | G | 41 | 36 | 6.03E-49 | 0.0742 |
| chr1:87715655:Y | chr1 | 9E+07 | 9E+07 | 733 | 5 | C | 28 | 26 | T | 21 | 3 | 1.80E-08 | 0.075 |
| chr1:87716417:Y | chr1 | 9E+07 | 9E+07 | 302 | 6 | C | 8 | 8 | T | 7 | 0 | 0.00016 | 0.096 |
| chr1:101609122:Y | chr1 | 1E+08 | 1E+08 | 331 | 5 | C | 26 | 25 | T | 11 | 4 | 0.00023 | 0 |
| chr1:108244475:Y | chr1 | 1E+08 | 1E+08 | 350 | 5 | C | 7 | 0 | T | 9 | 8 | 0.0007 | 0 |
| chr1:116952441:K | chr1 | 1E+08 | 1E+08 | 433 | 11 | G | 34 | 13 | T | 35 | 29 | 0.00019 | 0.0808 |
| chr1:141559129:Y | chr1 | 1E+08 | 1E+08 | 539 | 10 | C | 41 | 14 | T | 82 | 65 | 2.24E-06 | 0 |
| chr1:143244982:R | chr1 | 1E+08 | 1E+08 | 337 | 16 | A | 30 | 0 | G | 58 | 40 | 4.61E-11 | 0 |
| chr1:143245287:R | chr1 | 1E+08 | 1E+08 | 512 | 22 | A | 181 | 47 | G | 103 | 54 | 1.05E-05 | 0 |
| chr1:143803951:R | chr1 | 1E+08 | 1E+08 | 208 | 12 | A | 40 | 30 | G | 11 | 1 | 0.00012 | 0 |
| chr1:143808451:K | chr1 | 1E+08 | 1E+08 | 323 | 6 | G | 17 | 16 | T | 29 | 6 | 1.24E-06 | 0 |
| chr1:143808626:R | chr1 | 1E+08 | 1E+08 | 457 | 5 | A | 37 | 10 | G | 37 | 27 | 8.90E-05 | 0 |
| chr1:147121803:R | chr1 | 1E+08 | 1E+08 | 244 | 26 | A | 50 | 30 | G | 107 | 2 | 1.14E-16 | 0.0656 |
| chr1:150428541:W | chr1 | 2E+08 | 2E+08 | 208 | 21 | A | 74 | 7 | T | 15 | 8 | 0.00033 | 0 |
| chr1:155431647:K | chr1 | 2E+08 | 2E+08 | 304 | 22 | G | 42 | 22 | T | 26 | 1 | 1.90E-05 | 0 |
| chr1:155515528:R | chr1 | 2E+08 | 2E+08 | 304 | 17 | A | 67 | 56 | G | 56 | 17 | 1.74E-09 | 0.0428 |
| chr1:178471594:R | chr1 | 2E+08 | 2E+08 | 274 | 17 | A | 10 | 6 | G | 39 | 2 | 0.00036 | 0.0912 |
| chr1:216365899:Y | chr1 | 2E+08 | 2E+08 | 308 | 5 | C | 6 | 0 | T | 15 | 13 | 0.00052 | 0 |
| chr1:218825974:R | chr1 | 2E+08 | 2E+08 | 134 | 8 | A | 6 | 0 | G | 21 | 17 | 0.00071 | 0 |
| chr1:221315133:Y | chr1 | 2E+08 | 2E+08 | 240 | 6 | C | 6 | 6 | T | 11 | 1 | 0.00057 | 0 |
| chr1:226530871:K | chr1 | 2E+08 | 2E+08 | 207 | 16 | G | 28 | 19 | T | 25 | 2 | 1.19E-05 | 0 |
| chr1:226849866:Y | chr1 | 2E+08 | 2E+08 | 397 | 28 | C | 30 | 15 | T | 70 | 0 | 6.12E-10 | 0.0831 |
| chr1:227528424:K | chr1 | 2E+08 | 2E+08 | 324 | 5 | G | 32 | 3 | T | 13 | 11 | 2.36E-06 | 0 |
| chr1:240555037:M | chr1 | 2E+08 | 2E+08 | 269 | 5 | A | 17 | 2 | C | 39 | 30 | 1.26E-05 | 0.052 |
| chr1:242554221:K | chr1 | 2E+08 | 2E+08 | 278 | 6 | G | 16 | 1 | T | 16 | 14 | 6.84E-06 | 0.0072 |
| chr1:246167090:M | chr1 | 2E+08 | 2E+08 | 304 | 28 | A | 39 | 36 | C | 124 | 9 | 3.65E-24 | 0 |
| chr1:246911582:Y | chr1 | 2E+08 | 2E+08 | 295 | 6 | C | 11 | 2 | T | 13 | 12 | 0.00052 | 0 |
| chr20:4090809:R | chr20 | 4E+06 | 4E+06 | 216 | 9 | A | 26 | 3 | G | 9 | 7 | 0.00053 | 0 |
| chr20:20785211:R | chr20 | 2E+07 | 2E+07 | 267 | 5 | A | 15 | 0 | G | 24 | 12 | 0.00091 | 0 |
| chr20:20866701:S | chr20 | 2E+07 | 2E+07 | 283 | 7 | C | 10 | 9 | G | 16 | 3 | 0.00078 | 0 |
| chr20:25681278:Y | chr20 | 3E+07 | 3E+07 | 458 | 5 | C | 66 | 46 | T | 66 | 15 | 5.37E-08 | 0 |
| chr20:25696285:R | chr20 | 3E+07 | 3E+07 | 820 | 5 | A | 55 | 13 | G | 81 | 46 | 0.00019 | 0.0317 |
| chr20:25782494:W | chr20 | 3E+07 | 3E+07 | 305 | 23 | A | 91 | 1 | T | 3 | 3 | 2.98E-05 | 0 |
| chr20:25787016:W | chr20 | 3E+07 | 3E+07 | 112 | 10 | A | 5 | 4 | T | 29 | 0 | 0.00011 | 0 |
| chr20:26020787:R | chr20 | 3E+07 | 3E+07 | 359 | 6 | A | 24 | 19 | G | 52 | 12 | 8.42E-06 | 0 |
| chr20:26137108:M | chr20 | 3E+07 | 3E+07 | 522 | 68 | A | 159 | 24 | C | ### | 2230 | 1.50E-07 | 0 |
| chr20:48335705:S | chr20 | 5E+07 | 5E+07 | 188 | 7 | C | 6 | 0 | G | 12 | 12 | 5.39E-05 | 0 |
| chr20:48499750:K | chr20 | 5E+07 | 5E+07 | 306 | 6 | G | 14 | 1 | T | 37 | 23 | 0.00044 | 0 |
| chr20:51910184:Y | chr20 | 5E+07 | 5E+07 | 596 | 6 | C | 31 | 28 | T | 6 | 1 | 0.00071 | 0.0604 |
| chr20:56849390:R | chr20 | 6E+07 | 6E+07 | 279 | 11 | A | 10 | 10 | G | 19 | 0 | 4.99E-08 | 0 |
| chr20:56851466:Y | chr20 | 6E+07 | 6E+07 | 321 | 7 | C | 11 | 0 | T | 5 | 5 | 0.00023 | 0 |
| chr20:56860527:Y | chr20 | 6E+07 | 6E+07 | 196 | 10 | C | 24 | 0 | T | 10 | 9 | 1.91E-07 | 0 |
| chr20:56863963:W | chr20 | 6E+07 | 6E+07 | 179 | 10 | A | 4 | 4 | T | 11 | 0 | 0.00073 | 0 |
| chr20:56896867:S | chr20 | 6E+07 | 6E+07 | 368 | 15 | C | 25 | 24 | G | 19 | 0 | 1.42E-11 | 0 |
| chr20:59337797:Y | chr20 | 6E+07 | 6E+07 | 249 | 6 | C | 8 | 2 | T | 19 | 18 | 0.00061 | 0.0643 |
| chr20:59948670:Y | chr20 | 6E+07 | 6E+07 | 278 | 11 | C | 32 | 28 | T | 18 | 7 | 0.0008 | 0.018 |
| chr20:60486775:M | chr20 | 6E+07 | 6E+07 | 304 | 15 | A | 22 | 1 | C | 12 | 10 | 5.12E-06 | 0.0132 |
| chr20:60990433:R | chr20 | 6E+07 | 6E+07 | 253 | 8 | A | 7 | 1 | G | 9 | 9 | 0.00087 | 0 |
| chr20:62329137:S | chr20 | 6E+07 | 6E+07 | 311 | 14 | C | 28 | 26 | G | 24 | 7 | 1.85E-06 | 0 |

| | | | | | | | | | | | | | |
|---|---|---|---|---|---|---|---|---|---|---|---|---|---|
| chr21:13666818:Y | chr21 | 1E+07 | 1E+07 | 202 | 9 | C | 9 | 2 | T | 19 | 17 | 0.00092 | 0.005 |
| chr21:14274743:Y | chr21 | 1E+07 | 1E+07 | 287 | 19 | C | 8 | 4 | T | 36 | 0 | 0.00052 | 0 |
| chr21:14370815:S | chr21 | 1E+07 | 1E+07 | 169 | 8 | C | 14 | 13 | G | 60 | 21 | 0.00014 | 0 |
| chr21:14378131:K | chr21 | 1E+07 | 1E+07 | 186 | 9 | G | 45 | 6 | T | 30 | 28 | 1.43E-12 | 0 |
| chr21:32167598:S | chr21 | 3E+07 | 3E+07 | 309 | 38 | C | 16 | 5 | G | 57 | 0 | 0.00029 | 0 |
| chr21:36221516:Y | chr21 | 4E+07 | 4E+07 | 252 | 5 | C | 21 | 20 | T | 8 | 2 | 0.00038 | 0 |
| chr21:38551067:R | chr21 | 4E+07 | 4E+07 | 259 | 8 | A | 25 | 8 | G | 23 | 20 | 0.00014 | 0 |
| chr21:42192603:M | chr21 | 4E+07 | 4E+07 | 368 | 10 | A | 34 | 10 | C | 56 | 2 | 0.0008 | 0.0978 |
| chr21:42765326:R | chr21 | 4E+07 | 4E+07 | 200 | 6 | A | 11 | 11 | G | 4 | 0 | 0.00073 | 0 |
| chr21:43446899:R | chr21 | 4E+07 | 4E+07 | 298 | 23 | A | 38 | 34 | G | 31 | 11 | 3.09E-06 | 0 |
| chr21:44530416:Y | chr21 | 4E+07 | 4E+07 | 187 | 17 | C | 13 | 3 | T | 10 | 10 | 0.00036 | 0 |
| chr21:45271289:S | chr21 | 5E+07 | 5E+07 | 228 | 9 | C | 18 | 17 | G | 6 | 1 | 0.00081 | 0 |
| chr21:45468901:R | chr21 | 5E+07 | 5E+07 | 264 | 11 | A | 8 | 0 | G | 40 | 29 | 0.0002 | 0 |
| chr21:46301154:Y | chr21 | 5E+07 | 5E+07 | 315 | 15 | C | 8 | 0 | T | 15 | 13 | 9.18E-05 | 0 |
| chr22:19109604:K | chr22 | 2E+07 | 2E+07 | 157 | 18 | G | 17 | 0 | T | 18 | 11 | 0.00011 | 0 |
| chr22:20622217:Y | chr22 | 2E+07 | 2E+07 | 290 | 14 | C | 22 | 17 | T | 9 | 0 | 9.93E-05 | 0 |
| chr22:22464394:Y | chr22 | 2E+07 | 2E+07 | 261 | 6 | C | 8 | 7 | T | 9 | 0 | 0.00041 | 0 |
| chr22:27405694:S | chr22 | 3E+07 | 3E+07 | 219 | 38 | C | 61 | 8 | G | 18 | 12 | 2.21E-05 | 0.0365 |
| chr22:28611138:R | chr22 | 3E+07 | 3E+07 | 654 | 5 | A | 24 | 21 | G | 14 | 4 | 0.00039 | 0 |
| chr22:35136197:M | chr22 | 4E+07 | 4E+07 | 228 | 17 | A | 64 | 3 | C | 54 | 18 | 5.80E-05 | 0 |
| chr22:44904458:Y | chr22 | 4E+07 | 4E+07 | 153 | 8 | C | 13 | 0 | T | 21 | 20 | 1.51E-08 | 0 |
| chr22:45238564:R | chr22 | 5E+07 | 5E+07 | 211 | 18 | A | 38 | 36 | G | 8 | 0 | 1.72E-07 | 0 |
| chr22:45375193:R | chr22 | 5E+07 | 5E+07 | 204 | 6 | A | 5 | 0 | G | 9 | 9 | 0.0005 | 0 |
| chr22:45576719:R | chr22 | 5E+07 | 5E+07 | 286 | 10 | A | 9 | 3 | G | 21 | 20 | 0.00088 | 0 |
| chr22:46166687:R | chr22 | 5E+07 | 5E+07 | 157 | 7 | A | 11 | 11 | G | 11 | 1 | 1.70E-05 | 0.0127 |
| chr22:46388819:Y | chr22 | 5E+07 | 5E+07 | 237 | 8 | C | 17 | 15 | T | 21 | 6 | 0.0003 | 0 |
| chr22:46984028:Y | chr22 | 5E+07 | 5E+07 | 195 | 12 | C | 5 | 0 | T | 26 | 22 | 0.00074 | 0 |
| chr22:47029127:Y | chr22 | 5E+07 | 5E+07 | 343 | 8 | C | 29 | 7 | T | 30 | 23 | 7.64E-05 | 0 |
| chr22:47435334:R | chr22 | 5E+07 | 5E+07 | 98 | 6 | A | 11 | 11 | G | 6 | 0 | 8.08E-05 | 0 |
| chr22:48261960:S | chr22 | 5E+07 | 5E+07 | 264 | 15 | C | 18 | 17 | G | 31 | 11 | 5.62E-05 | 0 |
| chr22:48380296:R | chr22 | 5E+07 | 5E+07 | 273 | 12 | A | 5 | 1 | G | 38 | 38 | 4.05E-05 | 0 |
| chr22:48412318:R | chr22 | 5E+07 | 5E+07 | 207 | 14 | A | 42 | 10 | G | 14 | 14 | 3.38E-07 | 0 |
| chr22:48436472:R | chr22 | 5E+07 | 5E+07 | 288 | 10 | A | 14 | 11 | G | 10 | 0 | 0.00015 | 0 |
| chr22:48815745:R | chr22 | 5E+07 | 5E+07 | 449 | 12 | A | 3 | 3 | G | 31 | 0 | 0.00017 | 0.0846 |
| chr22:49244892:R | chr22 | 5E+07 | 5E+07 | 112 | 12 | A | 9 | 8 | G | 9 | 0 | 0.00021 | 0 |
| chr2:626657:K | chr2 | 626525 | 626780 | 256 | 31 | G | 52 | 8 | T | 25 | 14 | 0.0004 | 0 |
| chr2:5687332:K | chr2 | 6E+06 | 6E+06 | 362 | 10 | G | 64 | 18 | T | 16 | 13 | 0.00028 | 0.0718 |
| chr2:8389446:S | chr2 | 8E+06 | 8E+06 | 280 | 9 | C | 23 | 9 | G | 11 | 11 | 0.00066 | 0 |
| chr2:8484336:R | chr2 | 8E+06 | 8E+06 | 331 | 8 | A | 20 | 17 | G | 21 | 4 | 3.21E-05 | 0 |
| chr2:8738035:M | chr2 | 9E+06 | 9E+06 | 316 | 6 | A | 13 | 5 | C | 19 | 18 | 0.0009 | 0 |
| chr2:10299466:R | chr2 | 1E+07 | 1E+07 | 162 | 7 | A | 19 | 1 | G | 26 | 22 | 7.33E-08 | 0 |
| chr2:10532797:R | chr2 | 1E+07 | 1E+07 | 315 | 13 | A | 13 | 0 | G | 25 | 22 | 1.03E-07 | 0 |
| chr2:11155600:Y | chr2 | 1E+07 | 1E+07 | 320 | 7 | C | 8 | 8 | T | 24 | 7 | 0.00061 | 0 |
| chr2:13643215:W | chr2 | 1E+07 | 1E+07 | 315 | 5 | A | 29 | 25 | T | 34 | 10 | 8.82E-06 | 0 |
| chr2:24950576:S | chr2 | 2E+07 | 2E+07 | 337 | 9 | C | 6 | 0 | G | 38 | 37 | 9.92E-07 | 0 |
| chr2:28621854:R | chr2 | 3E+07 | 3E+07 | 248 | 11 | A | 10 | 0 | G | 7 | 7 | 5.14E-05 | 0 |
| chr2:37527929:K | chr2 | 4E+07 | 4E+07 | 184 | 5 | G | 33 | 3 | T | 5 | 5 | 0.00011 | 0 |
| chr2:37819151:Y | chr2 | 4E+07 | 4E+07 | 766 | 5 | C | 35 | 24 | T | 31 | 3 | 9.77E-07 | 0.0013 |
| chr2:37829156:M | chr2 | 4E+07 | 4E+07 | 685 | 5 | A | 13 | 1 | C | 48 | 39 | 1.82E-06 | 0.0934 |
| chr2:37854744:M | chr2 | 4E+07 | 4E+07 | 1134 | 6 | A | 61 | 49 | C | 22 | 4 | 3.85E-07 | 0.0608 |
| chr2:62334962:R | chr2 | 6E+07 | 6E+07 | 259 | 8 | A | 9 | 9 | G | 8 | 1 | 0.00041 | 0 |

| | | | | | | | | | | | | | |
|---|---|---|---|---|---|---|---|---|---|---|---|---|---|
| chr2:63505500:R | chr2 | 6E+07 | 6E+07 | 233 | 9 | A | 6 | 0 | G | 23 | 19 | 0.00044 | 0 |
| chr2:71059484:R | chr2 | 7E+07 | 7E+07 | 273 | 18 | A | 36 | 0 | G | 29 | 13 | 4.13E-06 | 0.0073 |
| chr2:75034905:Y | chr2 | 8E+07 | 8E+07 | 253 | 5 | C | 40 | 24 | T | 21 | 3 | 0.00092 | 0 |
| chr2:81747216:K | chr2 | 8E+07 | 8E+07 | 366 | 8 | G | 131 | 20 | T | 16 | 15 | 3.77E-10 | 0.0628 |
| chr2:85007336:M | chr2 | 9E+07 | 9E+07 | 306 | 15 | A | 19 | 11 | C | 36 | 1 | 6.32E-06 | 0.0948 |
| chr2:90998991:Y | chr2 | 9E+07 | 9E+07 | 249 | 25 | C | 9 | 7 | T | 113 | 2 | 1.88E-08 | 0.0643 |
| chr2:90999034:R | chr2 | 9E+07 | 9E+07 | 239 | 22 | A | 24 | 3 | G | 56 | 31 | 0.00043 | 0.0669 |
| chr2:94890883:S | chr2 | 9E+07 | 9E+07 | 474 | 34 | C | 85 | 16 | G | 171 | 111 | 1.69E-12 | 0 |
| chr2:94899427:R | chr2 | 9E+07 | 9E+07 | 432 | 10 | A | 36 | 11 | G | 27 | 22 | 9.73E-05 | 0 |
| chr2:94903947:M | chr2 | 9E+07 | 9E+07 | 278 | 13 | A | 62 | 40 | C | 42 | 5 | 7.19E-08 | 0 |
| chr2:94910982:W | chr2 | 9E+07 | 9E+07 | 487 | 5 | A | 29 | 19 | T | 33 | 6 | 0.00023 | 0.0575 |
| chr2:94915484:K | chr2 | 9E+07 | 9E+07 | 443 | 6 | G | 170 | 54 | T | 41 | 32 | 9.15E-08 | 0.079 |
| chr2:95971190:S | chr2 | 1E+08 | 1E+08 | 448 | 5 | C | 105 | 70 | G | 178 | 30 | 3.56E-17 | 0 |
| chr2:97274706:R | chr2 | 1E+08 | 1E+08 | 375 | 6 | A | 22 | 19 | G | 106 | 12 | 7.09E-12 | 0 |
| chr2:100951327:Y | chr2 | 1E+08 | 1E+08 | 106 | 8 | C | 12 | 11 | T | 5 | 0 | 0.00097 | 0 |
| chr2:104642363:S | chr2 | 1E+08 | 1E+08 | 238 | 18 | C | 20 | 16 | G | 35 | 0 | 1.63E-10 | 0 |
| chr2:109113174:W | chr2 | 1E+08 | 1E+08 | 184 | 13 | A | 20 | 0 | T | 10 | 10 | 3.33E-08 | 0 |
| chr2:129261990:R | chr2 | 1E+08 | 1E+08 | 244 | 6 | A | 17 | 14 | G | 10 | 1 | 0.00075 | 0 |
| chr2:130381133:M | chr2 | 1E+08 | 1E+08 | 315 | 8 | A | 15 | 2 | C | 36 | 24 | 0.00065 | 0 |
| chr2:130480085:S | chr2 | 1E+08 | 1E+08 | 243 | 28 | C | 50 | 0 | G | 30 | 8 | 0.0002 | 0 |
| chr2:132727352:M | chr2 | 1E+08 | 1E+08 | 329 | 12 | A | 33 | 28 | C | 257 | 94 | 1.13E-07 | 0 |
| chr2:132728084:R | chr2 | 1E+08 | 1E+08 | 364 | 16 | A | 96 | 28 | G | 25 | 21 | 1.11E-06 | 0 |
| chr2:132730409:M | chr2 | 1E+08 | 1E+08 | 220 | 23 | A | 17 | 0 | C | 29 | 14 | 0.00052 | 0 |
| chr2:132731578:R | chr2 | 1E+08 | 1E+08 | 320 | 29 | A | 29 | 0 | G | 617 | 266 | 1.72E-07 | 0 |
| chr2:132736986:M | chr2 | 1E+08 | 1E+08 | 518 | 8 | A | 110 | 44 | C | ### | 1947 | 1.57E-20 | 0 |
| chr2:132742036:M | chr2 | 1E+08 | 1E+08 | 478 | 24 | A | 63 | 7 | C | ### | 888 | 3.64E-08 | 0 |
| chr2:132778841:S | chr2 | 1E+08 | 1E+08 | 786 | 25 | C | 95 | 49 | G | 177 | 36 | 2.46E-07 | 0 |
| chr2:193255752:M | chr2 | 2E+08 | 2E+08 | 127 | 9 | A | 20 | 19 | C | 6 | 0 | 3.04E-05 | 0.0394 |
| chr2:206824960:M | chr2 | 2E+08 | 2E+08 | 383 | 7 | A | 29 | 4 | C | 21 | 17 | 2.25E-06 | 0 |
| chr2:218895611:Y | chr2 | 2E+08 | 2E+08 | 169 | 8 | C | 14 | 14 | T | 10 | 3 | 0.00035 | 0 |
| chr2:231763397:W | chr2 | 2E+08 | 2E+08 | 289 | 13 | A | 46 | 38 | T | 21 | 7 | 0.00015 | 0 |
| chr2:235064401:S | chr2 | 2E+08 | 2E+08 | 282 | 10 | C | 30 | 9 | G | 49 | 0 | 6.95E-05 | 0 |
| chr2:236284095:R | chr2 | 2E+08 | 2E+08 | 256 | 11 | A | 4 | 0 | G | 12 | 12 | 0.00055 | 0 |
| chr2:238479562:M | chr2 | 2E+08 | 2E+08 | 286 | 9 | A | 19 | 16 | C | 28 | 8 | 0.00028 | 0.0315 |
| chr2:239649115:Y | chr2 | 2E+08 | 2E+08 | 293 | 9 | C | 29 | 22 | T | 15 | 3 | 0.00092 | 0 |
| chr2:239932430:Y | chr2 | 2E+08 | 2E+08 | 261 | 6 | C | 16 | 16 | T | 9 | 2 | 7.49E-05 | 0 |
| chr2:240520543:Y | chr2 | 2E+08 | 2E+08 | 205 | 8 | C | 11 | 2 | T | 18 | 17 | 5.00E-05 | 0.0195 |
| chr2:240756355:R | chr2 | 2E+08 | 2E+08 | 185 | 11 | A | 14 | 5 | G | 15 | 15 | 0.0002 | 0 |
| chr2:241356473:Y | chr2 | 2E+08 | 2E+08 | 125 | 6 | C | 8 | 8 | T | 5 | 0 | 0.00078 | 0 |
| chr2:241699664:M | chr2 | 2E+08 | 2E+08 | 285 | 7 | A | 10 | 8 | C | 9 | 0 | 0.00071 | 0 |
| chr2:241706887:R | chr2 | 2E+08 | 2E+08 | 231 | 9 | A | 43 | 41 | G | 4 | 0 | 8.41E-05 | 0 |
| chr2:242083482:K | chr2 | 2E+08 | 2E+08 | 244 | 9 | G | 13 | 0 | T | 6 | 6 | 3.69E-05 | 0 |
| chr2:242128954:S | chr2 | 2E+08 | 2E+08 | 186 | 10 | C | 10 | 0 | G | 7 | 7 | 5.14E-05 | 0 |
| chr3:13220673:S | chr3 | 1E+07 | 1E+07 | 238 | 20 | C | 39 | 3 | G | 12 | 9 | 1.30E-05 | 0 |
| chr3:14579331:Y | chr3 | 1E+07 | 1E+07 | 124 | 6 | C | 13 | 12 | T | 6 | 0 | 0.00026 | 0 |
| chr3:16221473:Y | chr3 | 2E+07 | 2E+07 | 121 | 8 | C | 8 | 0 | T | 5 | 5 | 0.00078 | 0 |
| chr3:16271672:S | chr3 | 2E+07 | 2E+07 | 351 | 5 | C | 9 | 9 | G | 10 | 1 | 0.00012 | 0 |
| chr3:58547617:S | chr3 | 6E+07 | 6E+07 | 232 | 21 | C | 53 | 6 | G | 26 | 13 | 0.00044 | 0.0129 |
| chr3:73242457:S | chr3 | 7E+07 | 7E+07 | 558 | 30 | C | 203 | 126 | G | ### | 217 | 1.29E-74 | 0.0215 |
| chr3:75766230:R | chr3 | 8E+07 | 8E+07 | 682 | 5 | A | 39 | 7 | G | 11 | 9 | 0.00018 | 0.022 |
| chr3:75774842:W | chr3 | 8E+07 | 8E+07 | 361 | 6 | A | 75 | 29 | T | 190 | 30 | 0.00013 | 0 |

| | | | | | | | | | | | | | | |
|---|---|---|---|---|---|---|---|---|---|---|---|---|---|---|
| chr3:75775304:M | chr3 | 8E+07 | 8E+07 | 456 | 9 | A | 243 | 103 | C | 54 | 8 | 9.06E-05 | 0 |
| chr3:75790617:R | chr3 | 8E+07 | 8E+07 | 328 | 14 | A | 68 | 9 | G | 54 | 25 | 8.24E-05 | 0 |
| chr3:75801884:Y | chr3 | 8E+07 | 8E+07 | 530 | 28 | C | 46 | 5 | T | 434 | 171 | 7.60E-05 | 0.0981 |
| chr3:75804145:Y | chr3 | 8E+07 | 8E+07 | 276 | 18 | C | 23 | 15 | T | 22 | 1 | 2.32E-05 | 0.0399 |
| chr3:75804207:R | chr3 | 8E+07 | 8E+07 | 176 | 11 | A | 11 | 0 | G | 28 | 20 | 4.51E-05 | 0 |
| chr3:75804262:M | chr3 | 8E+07 | 8E+07 | 204 | 15 | A | 38 | 6 | C | 13 | 9 | 0.00067 | 0 |
| chr3:75804456:R | chr3 | 8E+07 | 8E+07 | 225 | 16 | A | 46 | 29 | G | 32 | 0 | 1.10E-09 | 0 |
| chr3:75876258:R | chr3 | 8E+07 | 8E+07 | 609 | 5 | A | 169 | 58 | G | 52 | 48 | 2.39E-14 | 0 |
| chr3:126143550:W | chr3 | 1E+08 | 1E+08 | 350 | 12 | A | 22 | 21 | T | 25 | 8 | 1.04E-05 | 0 |
| chr3:127191740:K | chr3 | 1E+08 | 1E+08 | 329 | 14 | G | 75 | 9 | T | 27 | 13 | 0.00024 | 0 |
| chr3:127573913:R | chr3 | 1E+08 | 1E+08 | 297 | 5 | A | 10 | 10 | G | 5 | 0 | 0.00033 | 0 |
| chr3:128423243:R | chr3 | 1E+08 | 1E+08 | 208 | 6 | A | 7 | 1 | G | 13 | 13 | 0.00018 | 0 |
| chr3:140145697:R | chr3 | 1E+08 | 1E+08 | 213 | 5 | A | 25 | 10 | G | 37 | 1 | 0.00025 | 0.0657 |
| chr3:195404878:M | chr3 | 2E+08 | 2E+08 | 130 | 15 | A | 9 | 5 | C | 27 | 0 | 0.00033 | 0 |
| chr3:196974738:S | chr3 | 2E+08 | 2E+08 | 276 | 24 | C | 35 | 28 | G | 13 | 0 | 4.02E-07 | 0 |
| chr3:197019179:M | chr3 | 2E+08 | 2E+08 | 276 | 10 | A | 32 | 25 | C | 20 | 3 | 1.08E-05 | 0 |
| chr4:1210862:Y | chr4 | 1E+06 | 1E+06 | 259 | 8 | C | 6 | 0 | T | 9 | 9 | 0.0002 | 0.0116 |
| chr4:2273521:S | chr4 | 2E+06 | 2E+06 | 149 | 8 | C | 24 | 20 | G | 6 | 0 | 0.00035 | 0 |
| chr4:2388243:S | chr4 | 2E+06 | 2E+06 | 295 | 19 | C | 65 | 19 | G | 44 | 0 | 1.73E-05 | 0 |
| chr4:3012982:K | chr4 | 3E+06 | 3E+06 | 212 | 15 | G | 14 | 13 | T | 20 | 4 | 5.91E-05 | 0 |
| chr4:3598518:R | chr4 | 4E+06 | 4E+06 | 965 | 5 | A | 41 | 14 | G | 44 | 34 | 7.66E-05 | 0 |
| chr4:3604962:S | chr4 | 4E+06 | 4E+06 | 448 | 9 | C | 21 | 8 | G | 25 | 22 | 0.00058 | 0.0335 |
| chr4:4278909:R | chr4 | 4E+06 | 4E+06 | 448 | 23 | A | 97 | 9 | G | 219 | 64 | 7.45E-05 | 0.0558 |
| chr4:4279357:K | chr4 | 4E+06 | 4E+06 | 294 | 34 | G | 46 | 7 | T | 88 | 41 | 0.00029 | 0 |
| chr4:7430406:M | chr4 | 7E+06 | 7E+06 | 331 | 8 | A | 21 | 11 | C | 16 | 0 | 0.00061 | 0 |
| chr4:12251123:K | chr4 | 1E+07 | 1E+07 | 359 | 9 | G | 94 | 5 | T | 17 | 15 | 1.40E-12 | 0 |
| chr4:12251243:Y | chr4 | 1E+07 | 1E+07 | 305 | 8 | C | 281 | 0 | T | 13 | 10 | 2.51E-16 | 0 |
| chr4:12251314:R | chr4 | 1E+07 | 1E+07 | 250 | 7 | A | 13 | 11 | G | 558 | 6 | 1.98E-17 | 0 |
| chr4:17047695:Y | chr4 | 2E+07 | 2E+07 | 241 | 13 | C | 11 | 0 | T | 11 | 11 | 2.84E-06 | 0 |
| chr4:33536356:M | chr4 | 3E+07 | 3E+07 | 317 | 5 | A | 15 | 2 | C | 38 | 34 | 2.44E-07 | 0 |
| chr4:40913388:Y | chr4 | 4E+07 | 4E+07 | 196 | 19 | C | 9 | 5 | T | 54 | 0 | 1.79E-05 | 0 |
| chr4:88411275:Y | chr4 | 9E+07 | 9E+07 | 253 | 7 | C | 9 | 8 | T | 8 | 0 | 0.00041 | 0 |
| chr4:106772696:R | chr4 | 1E+08 | 1E+08 | 481 | 6 | A | 10 | 10 | G | 19 | 4 | 5.00E-05 | 0 |
| chr4:113705494:M | chr4 | 1E+08 | 1E+08 | 280 | 12 | A | 29 | 28 | C | 14 | 1 | 5.19E-09 | 0 |
| chr4:152549944:S | chr4 | 2E+08 | 2E+08 | 256 | 17 | C | 7 | 5 | G | 29 | 0 | 5.57E-05 | 0.0391 |
| chr4:154932647:K | chr4 | 2E+08 | 2E+08 | 352 | 12 | G | 15 | 9 | T | 14 | 0 | 0.0007 | 0 |
| chr4:170006774:R | chr4 | 2E+08 | 2E+08 | 271 | 5 | A | 19 | 13 | G | 9 | 0 | 0.00083 | 0 |
| chr4:178629391:Y | chr4 | 2E+08 | 2E+08 | 188 | 14 | C | 12 | 2 | T | 30 | 27 | 1.07E-05 | 0.0798 |
| chr4:184048481:R | chr4 | 2E+08 | 2E+08 | 301 | 5 | A | 19 | 9 | G | 23 | 23 | 6.28E-05 | 0 |
| chr4:185235203:W | chr4 | 2E+08 | 2E+08 | 337 | 5 | A | 14 | 0 | T | 17 | 12 | 4.45E-05 | 0 |
| chr4:185448266:M | chr4 | 2E+08 | 2E+08 | 295 | 7 | A | 23 | 21 | C | 18 | 6 | 0.00016 | 0 |
| chr4:190437653:Y | chr4 | 2E+08 | 2E+08 | 392 | 11 | C | 32 | 19 | T | 137 | 28 | 3.60E-05 | 0.0765 |
| chr4:190847738:R | chr4 | 2E+08 | 2E+08 | 516 | 7 | A | 76 | 22 | G | 34 | 28 | 2.53E-07 | 0 |
| chr4:191098899:R | chr4 | 2E+08 | 2E+08 | 418 | 36 | A | 104 | 4 | G | 63 | 22 | 1.33E-07 | 0 |
| chr4:191138119:R | chr4 | 2E+08 | 2E+08 | 296 | 5 | A | 20 | 3 | G | 19 | 14 | 0.00033 | 0 |
| chr4:191146092:M | chr4 | 2E+08 | 2E+08 | 826 | 6 | A | 99 | 23 | C | 59 | 35 | 7.63E-06 | 0.0569 |
| chr4:191173084:S | chr4 | 2E+08 | 2E+08 | 340 | 37 | C | 200 | 107 | G | 104 | 20 | 5.40E-09 | 0.0471 |
| chr4:191177438:R | chr4 | 2E+08 | 2E+08 | 340 | 32 | A | 30 | 0 | G | 87 | 33 | 1.00E-05 | 0.0088 |
| chr5:1140868:Y | chr5 | 1E+06 | 1E+06 | 213 | 11 | C | 6 | 0 | T | 13 | 13 | 3.69E-05 | 0 |
| chr5:1647879:S | chr5 | 2E+06 | 2E+06 | 318 | 30 | C | 36 | 0 | G | 31 | 13 | 8.13E-06 | 0 |
| chr5:1940200:S | chr5 | 2E+06 | 2E+06 | 307 | 49 | C | 32 | 0 | G | 50 | 17 | 0.00012 | 0 |

| | | | | | | | | | | | | | |
|---|---|---|---|---|---|---|---|---|---|---|---|---|---|
| chr5:2002044:M | chr5 | 2E+06 | 2E+06 | 107 | 9 | A | 4 | 0 | C | 13 | 13 | 0.00042 | 0 |
| chr5:7342949:S | chr5 | 7E+06 | 7E+06 | 189 | 18 | C | 36 | 35 | G | 5 | 0 | 8.01E-06 | 0 |
| chr5:7902989:S | chr5 | 8E+06 | 8E+06 | 319 | 18 | C | 56 | 16 | G | 45 | 0 | 3.85E-05 | 0 |
| chr5:9752519:Y | chr5 | 1E+07 | 1E+07 | 257 | 6 | C | 30 | 26 | T | 27 | 10 | 0.00013 | 0 |
| chr5:74309014:W | chr5 | 7E+07 | 7E+07 | 358 | 6 | A | 20 | 3 | T | 21 | 16 | 0.00013 | 0 |
| chr5:75612211:S | chr5 | 8E+07 | 8E+07 | 179 | 9 | C | 11 | 11 | G | 6 | 1 | 0.00097 | 0 |
| chr5:99415346:R | chr5 | 1E+08 | 1E+08 | 347 | 6 | A | 11 | 11 | G | 112 | 4 | 8.86E-13 | 0.0807 |
| chr5:99416069:R | chr5 | 1E+08 | 1E+08 | 446 | 9 | A | 20 | 13 | G | 718 | 38 | 9.35E-12 | 0 |
| chr5:99416970:R | chr5 | 1E+08 | 1E+08 | 354 | 9 | A | 7 | 5 | G | 232 | 22 | 0.00023 | 0.096 |
| chr5:99417401:S | chr5 | 1E+08 | 1E+08 | 352 | 9 | C | 16 | 11 | G | 168 | 22 | 2.99E-06 | 0 |
| chr5:118719592:M | chr5 | 1E+08 | 1E+08 | 185 | 16 | A | 47 | 0 | C | 19 | 11 | 7.04E-08 | 0 |
| chr5:134287366:R | chr5 | 1E+08 | 1E+08 | 554 | 19 | A | ### | 56 | G | 110 | 83 | 3.55E-87 | 0.0975 |
| chr5:134287698:Y | chr5 | 1E+08 | 1E+08 | 257 | 6 | C | 8 | 5 | T | 114 | 0 | 2.70E-07 | 0 |
| chr5:134288288:R | chr5 | 1E+08 | 1E+08 | 374 | 7 | A | 28 | 19 | G | 208 | 9 | 6.94E-15 | 0 |
| chr5:134288729:R | chr5 | 1E+08 | 1E+08 | 352 | 11 | A | 403 | 7 | G | 44 | 32 | 3.37E-32 | 0 |
| chr5:134288945:R | chr5 | 1E+08 | 1E+08 | 383 | 10 | A | 52 | 38 | G | 400 | 2 | 4.25E-41 | 0 |
| chr5:134289274:R | chr5 | 1E+08 | 1E+08 | 355 | 5 | A | 230 | 3 | G | 13 | 8 | 7.47E-10 | 0 |
| chr5:134290558:R | chr5 | 1E+08 | 1E+08 | 436 | 12 | A | 782 | 8 | G | 66 | 38 | 4.32E-40 | 0 |
| chr5:134290858:R | chr5 | 1E+08 | 1E+08 | 437 | 12 | A | 39 | 29 | G | 567 | 6 | 3.31E-35 | 0 |
| chr5:134291008:Y | chr5 | 1E+08 | 1E+08 | 534 | 11 | C | 805 | 9 | T | 41 | 36 | 2.05E-49 | 0 |
| chr5:134291300:R | chr5 | 1E+08 | 1E+08 | 383 | 8 | A | 20 | 15 | G | 363 | 14 | 3.09E-15 | 0 |
| chr5:134291910:R | chr5 | 1E+08 | 1E+08 | 420 | 7 | A | 33 | 28 | G | 422 | 2 | 2.70E-37 | 0 |
| chr5:135442963:R | chr5 | 1E+08 | 1E+08 | 314 | 16 | A | 22 | 5 | G | 22 | 21 | 5.77E-07 | 0 |
| chr5:135443199:M | chr5 | 1E+08 | 1E+08 | 285 | 11 | A | 11 | 9 | C | 13 | 1 | 0.00052 | 0 |
| chr5:135443625:R | chr5 | 1E+08 | 1E+08 | 366 | 14 | A | 62 | 2 | G | 41 | 35 | 6.47E-19 | 0 |
| chr5:162536578:Y | chr5 | 2E+08 | 2E+08 | 268 | 8 | C | 12 | 2 | T | 9 | 9 | 0.00022 | 0.0224 |
| chr5:178383601:Y | chr5 | 2E+08 | 2E+08 | 586 | 34 | C | 75 | 10 | T | 71 | 31 | 4.74E-05 | 0.087 |
| chr5:178919042:R | chr5 | 2E+08 | 2E+08 | 273 | 13 | A | 14 | 7 | G | 25 | 0 | 0.00022 | 0 |
| chr5:178919238:M | chr5 | 2E+08 | 2E+08 | 266 | 16 | A | 21 | 0 | C | 10 | 9 | 4.96E-07 | 0 |
| chr5:179978950:S | chr5 | 2E+08 | 2E+08 | 254 | 16 | C | 10 | 1 | G | 12 | 10 | 0.00097 | 0 |
| chr6:236744:S | chr6 | 236624 | 236896 | 273 | 9 | C | 8 | 7 | G | 40 | 4 | 3.28E-05 | 0 |
| chr6:238274:M | chr6 | 238142 | 238442 | 301 | 10 | A | 15 | 9 | C | 48 | 0 | 2.11E-07 | 0 |
| chr6:261343:R | chr6 | 261202 | 261511 | 310 | 7 | A | 6 | 0 | G | 35 | 32 | 1.87E-05 | 0 |
| chr6:6559333:M | chr6 | 7E+06 | 7E+06 | 317 | 8 | A | 10 | 10 | C | 9 | 2 | 0.00071 | 0 |
| chr6:15205442:Y | chr6 | 2E+07 | 2E+07 | 278 | 5 | C | 13 | 11 | T | 12 | 1 | 0.00021 | 0 |
| chr6:25136045:Y | chr6 | 3E+07 | 3E+07 | 131 | 7 | C | 14 | 2 | T | 9 | 9 | 6.73E-05 | 0 |
| chr6:26413622:R | chr6 | 3E+07 | 3E+07 | 216 | 6 | A | 11 | 0 | G | 6 | 5 | 0.00097 | 0 |
| chr6:27306236:Y | chr6 | 3E+07 | 3E+07 | 329 | 15 | C | 30 | 10 | T | 29 | 0 | 0.0008 | 0 |
| chr6:28709775:R | chr6 | 3E+07 | 3E+07 | 264 | 9 | A | 15 | 3 | G | 20 | 16 | 0.00063 | 0 |
| chr6:29756098:R | chr6 | 3E+07 | 3E+07 | 256 | 7 | A | 10 | 9 | G | 7 | 0 | 0.00041 | 0 |
| chr6:29756543:Y | chr6 | 3E+07 | 3E+07 | 279 | 11 | C | 22 | 17 | T | 11 | 0 | 2.26E-05 | 0 |
| chr6:29904285:R | chr6 | 3E+07 | 3E+07 | 258 | 21 | A | 40 | 3 | G | 35 | 15 | 0.0004 | 0 |
| chr6:30001557:R | chr6 | 3E+07 | 3E+07 | 618 | 16 | A | 73 | 19 | G | 50 | 39 | 1.58E-08 | 0.0615 |
| chr6:30019298:K | chr6 | 3E+07 | 3E+07 | 165 | 10 | G | 21 | 0 | T | 9 | 7 | 1.77E-05 | 0.0182 |
| chr6:30052347:Y | chr6 | 3E+07 | 3E+07 | 224 | 5 | C | 3 | 3 | T | 20 | 0 | 0.00056 | 0 |
| chr6:30147077:Y | chr6 | 3E+07 | 3E+07 | 174 | 17 | C | 19 | 14 | T | 28 | 4 | 5.78E-05 | 0 |
| chr6:31663371:M | chr6 | 3E+07 | 3E+07 | 320 | 7 | A | 7 | 7 | C | 12 | 2 | 0.00071 | 0 |
| chr6:32651040:S | chr6 | 3E+07 | 3E+07 | 342 | 6 | C | 17 | 4 | G | 50 | 39 | 9.89E-05 | 0 |
| chr6:32836871:R | chr6 | 3E+07 | 3E+07 | 296 | 9 | A | 41 | 36 | G | 8 | 0 | 2.85E-06 | 0 |
| chr6:32836920:R | chr6 | 3E+07 | 3E+07 | 210 | 8 | A | 5 | 0 | G | 23 | 20 | 0.00057 | 0 |
| chr6:32836924:Y | chr6 | 3E+07 | 3E+07 | 237 | 11 | C | 25 | 22 | T | 5 | 0 | 0.00039 | 0 |

| | | | | | | | | | | | | | | |
|---|---|---|---|---|---|---|---|---|---|---|---|---|---|---|
| chr6:36845477:R | chr6 | 4E+07 | 4E+07 | 183 | 12 | A | 29 | 2 | G | 8 | 7 | 2.63E-05 | 0 |
| chr6:37591691:Y | chr6 | 4E+07 | 4E+07 | 246 | 7 | C | 30 | 5 | T | 14 | 12 | 1.95E-05 | 0 |
| chr6:42152923:R | chr6 | 4E+07 | 4E+07 | 217 | 9 | A | 6 | 1 | G | 15 | 15 | 0.00029 | 0 |
| chr6:44066125:Y | chr6 | 4E+07 | 4E+07 | 266 | 6 | C | 7 | 0 | T | 18 | 14 | 0.00069 | 0 |
| chr6:100173274:R | chr6 | 1E+08 | 1E+08 | 236 | 18 | A | 24 | 15 | G | 14 | 0 | 9.67E-05 | 0 |
| chr6:136652565:M | chr6 | 1E+08 | 1E+08 | 334 | 29 | A | 35 | 28 | C | 52 | 0 | 1.35E-16 | 0 |
| chr6:136652610:Y | chr6 | 1E+08 | 1E+08 | 225 | 23 | C | 63 | 0 | T | 11 | 11 | 2.39E-13 | 0 |
| chr6:150112222:S | chr6 | 2E+08 | 2E+08 | 315 | 30 | C | 43 | 15 | G | 61 | 0 | 3.18E-07 | 0 |
| chr6:150345094:Y | chr6 | 2E+08 | 2E+08 | 476 | 5 | C | 20 | 14 | T | 24 | 3 | 0.00014 | 0.0735 |
| chr6:158790069:R | chr6 | 2E+08 | 2E+08 | 294 | 13 | A | 20 | 10 | G | 18 | 18 | 0.00048 | 0 |
| chr6:158985741:S | chr6 | 2E+08 | 2E+08 | 166 | 18 | C | 18 | 6 | G | 44 | 0 | 0.0003 | 0 |
| chr6:160605062:Y | chr6 | 2E+08 | 2E+08 | 230 | 5 | C | 17 | 17 | T | 13 | 5 | 0.00022 | 0 |
| chr6:161186012:K | chr6 | 2E+08 | 2E+08 | 298 | 5 | G | 70 | 54 | T | 9 | 0 | 9.93E-06 | 0.0034 |
| chr6:163959633:R | chr6 | 2E+08 | 2E+08 | 248 | 8 | A | 13 | 13 | G | 14 | 4 | 0.00015 | 0 |
| chr6:166780986:R | chr6 | 2E+08 | 2E+08 | 197 | 5 | A | 23 | 21 | G | 8 | 2 | 0.00092 | 0 |
| chr6:167427499:R | chr6 | 2E+08 | 2E+08 | 86 | 6 | A | 6 | 0 | G | 11 | 10 | 0.00057 | 0 |
| chr6:170255518:Y | chr6 | 2E+08 | 2E+08 | 316 | 12 | C | 7 | 2 | T | 17 | 17 | 0.00049 | 0 |
| chr7:281549:Y | chr7 | 281488 | 281661 | 174 | 12 | C | 12 | 12 | T | 5 | 0 | 0.00016 | 0 |
| chr7:576303:R | chr7 | 576213 | 576385 | 173 | 11 | A | 20 | 19 | G | 4 | 0 | 0.00047 | 0 |
| chr7:592504:Y | chr7 | 592376 | 592639 | 264 | 7 | C | 6 | 1 | T | 13 | 13 | 0.00052 | 0 |
| chr7:1589301:S | chr7 | 2E+06 | 2E+06 | 317 | 7 | C | 20 | 19 | G | 29 | 13 | 0.00024 | 0 |
| chr7:2609626:R | chr7 | 3E+06 | 3E+06 | 267 | 9 | A | 25 | 23 | G | 6 | 1 | 0.00069 | 0 |
| chr7:19122981:S | chr7 | 2E+07 | 2E+07 | 330 | 36 | C | 61 | 11 | G | 114 | 2 | 0.0002 | 0.1 |
| chr7:27258445:W | chr7 | 3E+07 | 3E+07 | 202 | 13 | A | 22 | 0 | T | 11 | 10 | 1.19E-07 | 0 |
| chr7:32324582:Y | chr7 | 3E+07 | 3E+07 | 248 | 11 | C | 15 | 10 | T | 13 | 0 | 0.00025 | 0 |
| chr7:35268278:S | chr7 | 4E+07 | 4E+07 | 245 | 18 | C | 48 | 17 | G | 30 | 1 | 0.00078 | 0 |
| chr7:48934541:Y | chr7 | 5E+07 | 5E+07 | 197 | 13 | C | 16 | 1 | T | 31 | 26 | 2.82E-07 | 0 |
| chr7:53222458:Y | chr7 | 5E+07 | 5E+07 | 252 | 15 | C | 20 | 20 | T | 12 | 4 | 4.71E-05 | 0 |
| chr7:53222570:S | chr7 | 5E+07 | 5E+07 | 277 | 17 | C | 19 | 7 | G | 48 | 41 | 0.00017 | 0 |
| chr7:55379549:Y | chr7 | 6E+07 | 6E+07 | 193 | 13 | C | 19 | 19 | T | 14 | 6 | 0.00022 | 0 |
| chr7:56054813:K | chr7 | 6E+07 | 6E+07 | 424 | 6 | G | 14 | 4 | T | 25 | 24 | 1.51E-05 | 0 |
| chr7:57664941:Y | chr7 | 6E+07 | 6E+07 | 420 | 5 | C | 42 | 11 | T | 21 | 16 | 0.00031 | 0 |
| chr7:57718828:M | chr7 | 6E+07 | 6E+07 | 341 | 13 | A | 44 | 35 | C | 17 | 5 | 0.00056 | 0.0587 |
| chr7:63135705:S | chr7 | 6E+07 | 6E+07 | 343 | 18 | C | 42 | 31 | G | 28 | 5 | 6.36E-06 | 0.0758 |
| chr7:65607963:M | chr7 | 7E+07 | 7E+07 | 250 | 13 | A | 38 | 18 | C | 46 | 44 | 4.69E-07 | 0 |
| chr7:100395430:M | chr7 | 1E+08 | 1E+08 | 374 | 21 | A | 123 | 32 | C | 45 | 1 | 0.00029 | 0 |
| chr7:102874071:K | chr7 | 1E+08 | 1E+08 | 280 | 19 | G | 50 | 3 | T | 5 | 5 | 1.61E-05 | 0 |
| chr7:105383950:R | chr7 | 1E+08 | 1E+08 | 142 | 10 | A | 6 | 0 | G | 18 | 17 | 5.20E-05 | 0.0986 |
| chr7:134513864:Y | chr7 | 1E+08 | 1E+08 | 182 | 8 | C | 8 | 8 | T | 5 | 0 | 0.00078 | 0 |
| chr7:135274877:S | chr7 | 1E+08 | 1E+08 | 277 | 5 | C | 8 | 7 | G | 20 | 0 | 6.76E-06 | 0.0686 |
| chr7:139772308:R | chr7 | 1E+08 | 1E+08 | 265 | 10 | A | 8 | 0 | G | 15 | 14 | 1.84E-05 | 0 |
| chr7:150081412:R | chr7 | 2E+08 | 2E+08 | 281 | 5 | A | 19 | 17 | G | 11 | 2 | 0.00018 | 0 |
| chr7:153522774:Y | chr7 | 2E+08 | 2E+08 | 312 | 7 | C | 19 | 7 | T | 24 | 21 | 0.00098 | 0.0128 |
| chr7:156907915:Y | chr7 | 2E+08 | 2E+08 | 188 | 10 | C | 7 | 7 | T | 11 | 1 | 0.00025 | 0 |
| chr7:157996235:R | chr7 | 2E+08 | 2E+08 | 282 | 7 | A | 18 | 7 | G | 26 | 24 | 0.00021 | 0 |
| chr7:158482807:M | chr7 | 2E+08 | 2E+08 | 319 | 20 | A | 22 | 1 | C | 30 | 25 | 6.43E-09 | 0 |
| chr7:158603416:Y | chr7 | 2E+08 | 2E+08 | 331 | 8 | C | 5 | 0 | T | 8 | 8 | 0.00078 | 0 |
| chr8:169693:K | chr8 | 169644 | 169776 | 133 | 14 | G | 15 | 15 | T | 8 | 0 | 2.04E-06 | 0 |
| chr8:1141599:S | chr8 | 1E+06 | 1E+06 | 306 | 8 | C | 13 | 13 | G | 15 | 5 | 0.00025 | 0 |
| chr8:1630014:K | chr8 | 2E+06 | 2E+06 | 179 | 10 | G | 15 | 15 | T | 7 | 1 | 9.38E-05 | 0 |
| chr8:1637157:R | chr8 | 2E+06 | 2E+06 | 242 | 21 | A | 31 | 0 | G | 3 | 3 | 0.00017 | 0 |

| | | | | | | | | | | | | | | |
|---|---|---|---|---|---|---|---|---|---|---|---|---|---|---|
| chr8:1641097:R | chr8 | 2E+06 | 2E+06 | 331 | 12 | A | 14 | 7 | G | 20 | 20 | 0.00064 | 0 |
| chr8:2053369:M | chr8 | 2E+06 | 2E+06 | 286 | 7 | A | 15 | 4 | C | 14 | 13 | 0.00048 | 0 |
| chr8:2113807:W | chr8 | 2E+06 | 2E+06 | 223 | 9 | A | 20 | 8 | T | 13 | 13 | 0.00051 | 0 |
| chr8:2872872:R | chr8 | 3E+06 | 3E+06 | 284 | 8 | A | 5 | 0 | G | 8 | 8 | 0.00078 | 0 |
| chr8:5140329:Y | chr8 | 5E+06 | 5E+06 | 179 | 5 | C | 15 | 1 | T | 17 | 12 | 0.0003 | 0 |
| chr8:6779011:R | chr8 | 7E+06 | 7E+06 | 216 | 8 | A | 21 | 9 | G | 25 | 23 | 0.00041 | 0 |
| chr8:11743990:K | chr8 | 1E+07 | 1E+07 | 186 | 11 | G | 26 | 26 | T | 14 | 5 | 7.32E-06 | 0 |
| chr8:17396802:K | chr8 | 2E+07 | 2E+07 | 297 | 6 | G | 48 | 20 | T | 29 | 26 | 3.02E-05 | 0 |
| chr8:37111840:R | chr8 | 4E+07 | 4E+07 | 240 | 5 | A | 7 | 7 | G | 6 | 0 | 0.00058 | 0 |
| chr8:39392227:W | chr8 | 4E+07 | 4E+07 | 409 | 6 | A | 37 | 4 | T | 32 | 18 | 7.19E-05 | 0 |
| chr8:49247350:R | chr8 | 5E+07 | 5E+07 | 347 | 11 | A | 42 | 1 | G | 7 | 4 | 0.00078 | 0 |
| chr8:55541766:S | chr8 | 6E+07 | 6E+07 | 199 | 19 | C | 39 | 0 | G | 27 | 9 | 0.00013 | 0 |
| chr8:101246933:M | chr8 | 1E+08 | 1E+08 | 302 | 12 | A | 46 | 37 | C | 34 | 9 | 2.54E-06 | 0 |
| chr8:128958553:M | chr8 | 1E+08 | 1E+08 | 166 | 7 | A | 17 | 17 | C | 19 | 9 | 0.00044 | 0 |
| chr8:142230214:M | chr8 | 1E+08 | 1E+08 | 283 | 16 | A | 32 | 16 | C | 20 | 20 | 9.70E-05 | 0 |
| chr8:143654275:R | chr8 | 1E+08 | 1E+08 | 336 | 6 | A | 5 | 4 | G | 19 | 0 | 0.00047 | 0 |
| chr9:31201304:Y | chr9 | 3E+07 | 3E+07 | 186 | 7 | C | 9 | 8 | T | 14 | 0 | 1.84E-05 | 0 |
| chr9:33513364:Y | chr9 | 3E+07 | 3E+07 | 429 | 7 | C | 12 | 1 | T | 20 | 14 | 0.00095 | 0.014 |
| chr9:34642330:R | chr9 | 3E+07 | 3E+07 | 315 | 12 | A | 17 | 16 | G | 17 | 5 | 0.00012 | 0.0254 |
| chr9:38516765:S | chr9 | 4E+07 | 4E+07 | 191 | 27 | C | 30 | 2 | G | 9 | 8 | 6.21E-06 | 0 |
| chr9:66194664:Y | chr9 | 7E+07 | 7E+07 | 333 | 13 | C | 131 | 63 | T | 46 | 5 | 3.39E-06 | 0 |
| chr9:66195700:Y | chr9 | 7E+07 | 7E+07 | 310 | 25 | C | 86 | 59 | T | 85 | 16 | 3.85E-11 | 0 |
| chr9:66195881:S | chr9 | 7E+07 | 7E+07 | 429 | 32 | C | 116 | 18 | G | 238 | 108 | 1.76E-08 | 0 |
| chr9:66206159:S | chr9 | 7E+07 | 7E+07 | 365 | 6 | C | 82 | 36 | G | 11 | 11 | 0.0005 | 0.0274 |
| chr9:67903287:W | chr9 | 7E+07 | 7E+07 | 349 | 12 | A | 19 | 0 | T | 28 | 13 | 0.00048 | 0 |
| chr9:67945026:M | chr9 | 7E+07 | 7E+07 | 331 | 17 | A | 23 | 1 | C | 63 | 28 | 0.00027 | 0 |
| chr9:67945153:R | chr9 | 7E+07 | 7E+07 | 318 | 12 | A | 26 | 15 | G | 22 | 0 | 7.22E-06 | 0.022 |
| chr9:67945381:R | chr9 | 7E+07 | 7E+07 | 340 | 21 | A | 78 | 40 | G | 108 | 16 | 1.24E-07 | 0.0206 |
| chr9:67966129:M | chr9 | 7E+07 | 7E+07 | 742 | 7 | A | 200 | 127 | C | 58 | 11 | 1.70E-09 | 0 |
| chr9:69118593:W | chr9 | 7E+07 | 7E+07 | 332 | 5 | A | 39 | 24 | T | 23 | 3 | 0.0002 | 0.0512 |
| chr9:70112025:Y | chr9 | 7E+07 | 7E+07 | 261 | 21 | C | 49 | 2 | T | 42 | 35 | 7.52E-16 | 0 |
| chr9:70112717:Y | chr9 | 7E+07 | 7E+07 | 377 | 10 | C | 20 | 3 | T | 8 | 7 | 0.00071 | 0.0398 |
| chr9:93014234:R | chr9 | 9E+07 | 9E+07 | 346 | 5 | A | 9 | 2 | G | 16 | 16 | 7.49E-05 | 0 |
| chr9:94715557:M | chr9 | 9E+07 | 9E+07 | 348 | 18 | A | 29 | 12 | C | 29 | 26 | 0.00012 | 0 |
| chr9:95469224:R | chr9 | 1E+08 | 1E+08 | 346 | 13 | A | 49 | 44 | G | 11 | 3 | 6.32E-05 | 0.0347 |
| chr9:97278179:R | chr9 | 1E+08 | 1E+08 | 326 | 10 | A | 11 | 4 | G | 17 | 17 | 0.00028 | 0 |
| chr9:122118106:M | chr9 | 1E+08 | 1E+08 | 214 | 6 | A | 8 | 0 | C | 25 | 18 | 0.00046 | 0 |
| chr9:128224283:R | chr9 | 1E+08 | 1E+08 | 208 | 7 | A | 13 | 13 | G | 13 | 2 | 1.01E-05 | 0 |
| chr9:129266518:W | chr9 | 1E+08 | 1E+08 | 251 | 8 | A | 12 | 12 | T | 4 | 0 | 0.00055 | 0 |
| chr9:129871150:R | chr9 | 1E+08 | 1E+08 | 227 | 10 | A | 22 | 0 | G | 4 | 4 | 6.69E-05 | 0 |
| chr9:135074989:Y | chr9 | 1E+08 | 1E+08 | 267 | 12 | C | 9 | 1 | T | 18 | 16 | 0.00017 | 0 |
| chr9:136241230:M | chr9 | 1E+08 | 1E+08 | 271 | 5 | A | 10 | 2 | C | 9 | 9 | 0.00071 | 0 |
| chr9:137423212:S | chr9 | 1E+08 | 1E+08 | 209 | 11 | C | 17 | 17 | G | 11 | 4 | 0.00028 | 0 |
| chr9:138641818:Y | chr9 | 1E+08 | 1E+08 | 185 | 7 | C | 21 | 18 | T | 5 | 0 | 0.00085 | 0 |
| chr9:138835832:S | chr9 | 1E+08 | 1E+08 | 206 | 30 | C | 11 | 9 | G | 40 | 6 | 6.85E-05 | 0 |
