# Supplementary material for: The DNA Methylome of Human Peripheral Blood Mononuclear Cells
Source: PLoS Biol. 2010 Nov 9;8(11):e1000533. doi: 10.1371/journal.pbio.1000533 (PMC2976721; doi:10.1371/journal.pbio.1000533)
Supplement: Table S5 — Genes with hDMRs. (0.21 MB PDF) [file pbio.1000533.s017.pdf]

Table S5. Genes with hDMRs.

YH heterozygote	Accession number	Gene symbol	DMR location	overlap/distance (bp)
chr1:3133631:Y	NM_199454	PRDM16	intron	229
chr1:3133631:Y	NM_022114	PRDM16	intron	229
chr1:3336208:S	NM_199454	PRDM16	intron	198
chr1:3336208:S	NM_022114	PRDM16	intron	198
chr1:7425882:M	NM_015215	CAMTA1	intron	251
chr1:10633865:Y	NM_017766	CASZ1	intron	141
chr1:10633865:Y	NM_001079843	CASZ1	intron	141
chr1:15145768:K	NM_001017999	KIAA1026	intron	222
chr1:15145768:K	NM_001018001	KIAA1026	intron	222
chr1:15145768:K	NM_001018000	KIAA1026	intron	222
chr1:15145768:K	NM_015209	KIAA1026	intron	222
chr1:15145768:K	NM_201628	KIAA1026	intron	222
chr1:16264378:Y	NM_182623	FAM131C	intron	340
chr1:16891185:R	NR_026567	ESPNP	intron	285
chr1:16895697:R	NR_026567	ESPNP	exon,intron	327
chr1:18551953:Y	NM_032880	IGSF21	intron	205
chr1:21737466:Y	NM_000478	ALPL	intron	250
chr1:28295434:Y	NR_027268	LOC653566	exon	317
chr1:32040924:M	NM_144569	SPOCD1	intron	333
chr1:77209519:S	NM_030965	ST6GALNAC5	intron	445
chr1:108244475:Y	NM_006113	VAV3	intron	350
chr1:116952441:K	NM_001542	IGSF3	exon,intron	433
chr1:116952441:K	NM_001007237	IGSF3	exon,intron	433
chr1:143808451:K	NM_004892	SEC22B	intron	323
chr1:143808626:R	NM_004892	SEC22B	intron	457
chr1:178471594:R	NM_033343	LHX4	intron	274
chr1:218825974:R	NM_018650	MARK1	intron	134
chr1:226530871:K	NM_052843	OBSCN	exon	207
chr1:226530871:K	NM_001098623	OBSCN	exon	207
chr1:226849866:Y	NR_002834	DUSP5P	intron	397
chr1:227528424:K	NM_145257	C1orf96	intron	324
chr1:240555037:M	NM_152666	PLD5	intron	269
chr1:246167090:M	NM_175911	OR2L13	exon,intron	202
chr1:246911582:Y	NM_001004734	OR14I1	exon	295
chr10:1428597:M	NM_018702	ADARB2	intron	253
chr10:11278201:R	NM_001025076	CUGBP2	intron	191
chr10:11278201:R	NM_001083591	CUGBP2	intron	191
chr10:11278201:R	NM_006561	CUGBP2	intron	191
chr10:11278201:R	NM_001025077	CUGBP2	intron	191
chr10:46390045:R	NM_031912	SYT15	intron	290

chr10:46390045:R	NM_181519	SYT15	intron	290
chr10:49331990:S	NM_021226	ARHGAP22	exon,intron	350
chr10:97062308:M	NM_001034954	SORBS1	exon	263
chr10:97062308:M	NM_001034956	SORBS1	exon	263
chr10:97062308:M	NM_024991	SORBS1	exon	263
chr10:97062308:M	NM_015385	SORBS1	exon	263
chr10:97062308:M	NM_001034955	SORBS1	exon	263
chr10:97062308:M	NM_006434	SORBS1	exon	263
chr10:97062308:M	NM_001034957	SORBS1	exon	263
chr10:123862117:Y	NM_206862	TACC2	intron	318
chr10:123862117:Y	NM_206861	TACC2	intron	318
chr10:126346830:K	NM_014661	FAM53B	intron	265
chr10:126671779:K	NM_001083914	CTBP2	exon,intron	416
chr10:126671779:K	NM_022802	CTBP2	exon,intron	416
chr10:126671779:K	NM_001329	CTBP2	exon,intron	416
chr10:127574384:R	NM_145235	FANK1	upstream	506
chr10:127574499:R	NM_145235	FANK1	upstream	439
chr10:127574488:Y	NM_145235	FANK1	upstream	552
chr10:127574788:S	NM_145235	FANK1	upstream	96
chr10:127575080:M	NM_145235	FANK1	exon,intron	235
chr10:127575425:K	NM_145235	FANK1	intron	400
chr10:127604847:K	NM_145235	FANK1	intron	322
chr10:135192305:S	NM_000773	CYP2E1	intron	264
chr11:487359:R	NM_002939	RNH1	intron	165
chr11:487359:R	NM_203387	RNH1	intron	165
chr11:487359:R	NM_203386	RNH1	intron	165
chr11:487359:R	NM_203388	RNH1	intron	165
chr11:487359:R	NM_203384	RNH1	intron	165
chr11:487359:R	NM_203383	RNH1	intron	165
chr11:487359:R	NM_203385	RNH1	intron	165
chr11:487359:R	NM_203389	RNH1	intron	165
chr11:1007231:R	NM_005961	MUC6	exon	329
chr11:1620616:Y	NM_053005	HCCA2	intron	232
chr11:2126331:S	NM_001042376	INS-IGF2	intron	408
chr11:2126331:S	NM_016412	IGF2AS	exon	255
chr11:2126331:S	NR_003512	INS-IGF2	intron	408
chr11:2126331:S	NM_001007139	IGF2	intron	408
chr11:2787141:Y	NM_181798	KCNQ1	intron	258
chr11:2787141:Y	NM_000218	KCNQ1	intron	258
chr11:19941915:S	NM_145117	NAV2	intron	395
chr11:19941915:S	NM_182964	NAV2	intron	395
chr11:19941915:S	NM_001111018	NAV2	intron	395
chr11:61264985:R	NM_006133	DAGLA	intron	201

chr11:67174754:Y	NM_080658	ACY3	exon	47
chr11:113929139:Y	NM_152315	FAM55A	intron	702
chr11:122184121:Y	NM_032873	UBASH3B	exon,intron	208
chr12:380722:Y	NM_032358	CCDC77	upstream	74
chr12:380722:Y	NM_001130146	CCDC77	upstream	74
chr12:380722:Y	NM_001130148	CCDC77	intron	349
chr12:380722:Y	NM_001130147	CCDC77	intron	349
chr12:1330815:S	NM_178038	ERC1	intron	340
chr12:1330815:S	NM_178037	ERC1	intron	340
chr12:1330815:S	NM_015064	ERC1	intron	340
chr12:1330815:S	NM_178039	ERC1	intron	340
chr12:1330815:S	NM_178040	ERC1	intron	340
chr12:11062670:S	NM_006250	PRH1	intron	695
chr12:11062670:S	NM_001098538	PRR4	intron	695
chr12:11116497:M	NM_006250	PRH1	intron	407
chr12:11116497:M	NM_001098538	PRR4	intron	407
chr12:51377833:M	NM_175078	KRT77	exon,intron	236
chr12:56503583:S	NM_005730	CTDSP2	exon	294
chr12:56503965:K	NM_005730	CTDSP2	exon,intron	292
chr12:56509545:R	NM_005730	CTDSP2	exon,intron	340
chr12:110944530:K	NM_006817	ERP29	exon	305
chr12:110944530:K	NM_001034025	ERP29	exon	305
chr12:120099514:S	NM_002562	P2RX7	exon,intron	307
chr12:120840540:Y	NM_144668	WDR66	upstream	155
chr12:131103205:R	NM_015409	EP400	intron	183
chr12:131253028:K	NM_001122636	GALNT9	intron	270
chr12:131253028:K	NM_021808	GALNT9	intron	270
chr13:18541183:Y	NR_002801	DKFZp686A1627	intron	441
chr13:48793645:R	NM_030925	CAB39L	intron	280
chr13:48793645:R	NM_001079670	CAB39L	intron	280
chr13:97994122:M	NM_001032296	STK24	intron	212
chr13:99433607:R	NM_007129	ZIC2	intron	314
chr13:99954646:R	NM_001127692	PCCA	intron	212
chr13:99954646:R	NM_000282	PCCA	intron	212
chr13:100866569:K	NM_052867	NALCN	intron	102
chr13:107144419:K	NM_001080396	FAM155A	intron	434
chr13:110069271:R	NM_018210	CARKD	intron	244
chr13:113607931:S	NM_182614	FAM70B	intron	286
chr13:113909298:Y	NM_007368	RASA3	intron	295
chr13:114065598:S	NM_080687	UPF3A	exon,intron	188
chr13:114065598:S	NM_023011	UPF3A	exon,intron	188
chr14:22486303:M	NM_017815	C14orf94	exon,intron	405
chr14:23711366:Y	NM_001048205	REC8	exon	239

chr14:23711366:Y	NM_005132	REC8	exon,intron	239
chr14:32023501:W	NM_004274	AKAP6	intron	468
chr14:34254292:Y	NM_138638	CFL2	upstream	503
chr14:34254292:Y	NM_021914	CFL2	upstream	1464
chr14:64329980:K	NM_000347	SPTB	exon	202
chr14:64329980:K	NM_001024858	SPTB	exon	202
chr14:77449437:Y	NM_020421	ADCK1	intron	272
chr14:77449437:Y	NM_001142545	ADCK1	intron	272
chr14:78780542:Y	NM_004796	NRXN3	intron	281
chr14:100363281:M	NR_002766	MEG3	intron	202
chr14:100363281:M	NR_003530	MEG3	intron	202
chr14:100363281:M	NR_003531	MEG3	intron	202
chr14:103646201:R	NM_001080464	ASPG	intron	193
chr14:104779371:R	NM_001519	BRF1	intron	241
chr14:104779371:R	NM_145685	BRF1	intron	241
chr15:24518540:S	NM_000814	GABRB3	intron	307
chr15:24518540:S	NM_021912	GABRB3	intron	307
chr15:27183361:Y	NM_001130414	APBA2	intron	237
chr15:27183361:Y	NM_005503	APBA2	intron	237
chr15:29587002:R	NM_130901	OTUD7A	intron	193
chr15:32658265:M	NM_001023567	GOLGA8B	intron	259
chr15:49700263:R	NM_015263	DMXL2	intron	373
chr15:82348680:K	NM_207517	ADAMTSL3	intron	329
chr15:83752652:R	NM_006738	AKAP13	intron	333
chr15:83752652:R	NM_007200	AKAP13	intron	333
chr15:84112597:M	NM_022480	KLHL25	exon	193
chr15:88546567:R	NM_198925	SEMA4B	intron	180
chr15:88546567:R	NM_020210	SEMA4B	intron	180
chr15:97467811:S	NM_145728	SYNM	intron	319
chr15:97467811:S	NM_015286	SYNM	intron	319
chr16:534472:M	NM_005632	SOLH	intron	169
chr16:798095:R	NM_001013638	LOC388199	intron	168
chr16:920089:R	NM_022773	LMF1	intron	350
chr16:3490148:M	NM_015041	CLUAP1	upstream	748
chr16:4081729:Y	NM_001116	ADCY9	intron	219
chr16:6815929:Y	NM_001142334	A2BP1	intron	225
chr16:6815929:Y	NM_001142333	A2BP1	intron	225
chr16:6815929:Y	NM_018723	A2BP1	intron	225
chr16:10112679:W	NM_001134408	GRIN2A	intron	244
chr16:10112679:W	NM_001134407	GRIN2A	intron	244
chr16:10112679:W	NM_000833	GRIN2A	intron	244
chr16:28525472:Y	NM_001055	SULT1A1	exon,intron	265
chr16:28525472:Y	NM_177536	SULT1A1	exon,intron	265

chr16:28525472:Y	NM_177529	SULT1A1	exon,intron	265
chr16:28525472:Y	NM_177530	SULT1A1	exon,intron	265
chr16:28525472:Y	NM_177534	SULT1A1	exon,intron	265
chr16:31109809:R	NM_004960	FUS	exon,intron	329
chr16:69470887:R	NM_032821	HYDIN	exon,intron	238
chr16:69512693:R	NM_032821	HYDIN	exon,intron	214
chr16:69619885:Y	NM_017558	HYDIN	intron	415
chr16:69619885:Y	NM_032821	HYDIN	intron	415
chr16:79848365:Y	NM_017429	BCMO1	intron	293
chr16:87097703:M	NM_153813	ZFPM1	intron	212
chr16:87296901:R	NM_178841	RNF166	intron	243
chr16:87527804:K	NM_175931	CBFA2T3	intron	260
chr16:87527804:K	NM_005187	CBFA2T3	intron	260
chr17:84936:R	NM_006987	RPH3AL	intron	240
chr17:1477461:M	NM_152346	SLC43A2	intron	215
chr17:8943190:R	NM_004822	NTN1	intron	245
chr17:20723886:S	NM_001004306	CCDC144NL	intron	368
chr17:21154730:Y	NM_002756	MAP2K3	intron	363
chr17:21154730:Y	NM_145109	MAP2K3	intron	363
chr17:21749692:K	NM_203392	FAM27L	exon,intron	238
chr17:42621661:S	NM_001114091	CDC27	exon	152
chr17:42621661:S	NM_001256	CDC27	exon	152
chr17:45425740:R	NM_005220	DLX3	exon,intron	189
chr17:58044277:M	NM_006852	TLK2	exon	357
chr17:58044277:M	NM_001112707	TLK2	exon	357
chr17:71529149:K	NM_001988	EVPL	exon,intron	205
chr17:74710619:M	NM_001082575	HRNBP3	intron	333
chr17:75660805:R	NM_017950	CCDC40	intron	293
chr17:77041649:S	NM_001080519	BAHCC1	intron	181
chr18:12602345:Y	NM_001128626	SPIRE1	intron	444
chr18:12602345:Y	NM_001128627	SPIRE1	intron	444
chr18:12602345:Y	NM_020148	SPIRE1	intron	444
chr18:42590883:M	NM_013305	ST8SIA5	exon	296
chr18:45974364:K	NM_001080467	MYO5B	intron	250
chr18:72245281:R	NM_014643	ZNF516	intron	305
chr18:72947624:Y	NM_001025100	MBP	intron	361
chr18:72947624:Y	NM_001025101	MBP	intron	361
chr18:75373624:Y	NM_172389	NFATC1	intron	163
chr18:75373624:Y	NM_172387	NFATC1	intron	163
chr18:75373624:Y	NM_006162	NFATC1	intron	163
chr18:75373624:Y	NM_172388	NFATC1	intron	163
chr18:75774550:R	NM_025078	PQLC1	intron	115
chr19:3320572:K	NM_005597	NFIC	intron	254

chr19:3320572:K	NM_205843	NFIC	intron	254
chr19:12737964:S	NM_001100176	HOOK2	exon,intron	193
chr19:12737964:S	NM_013312	HOOK2	exon,intron	193
chr19:17458830:K	NM_198580	SLC27A1	exon,intron	156
chr19:18118750:Y	NM_015016	MAST3	exon,intron	170
chr19:37875509:M	NM_001105570	NUDT19	exon,intron	163
chr19:41335535:K	NM_001864	COX7A1	exon	172
chr19:44157852:W	NM_024907	FBXO17	intron	191
chr19:49345887:M	NM_006630	ZNF234	intron	311
chr19:49345887:M	NM_001144824	ZNF234	intron	311
chr19:50182410:R	NM_001294	CLPTM1	exon,intron	158
chr19:55104029:S	NM_016553	NUP62	exon	99
chr19:55104029:S	NM_012346	NUP62	exon	99
chr19:55104029:S	NM_153718	NUP62	exon	99
chr19:55104029:S	NM_153719	NUP62	exon	99
chr19:55104029:S	NM_172374	IL4I1	intron	99
chr19:62042275:Y	NR_024059	MIMT1	upstream	1651
chr19:62042275:Y	NM_006210	PEG3	intron	342
chr19:62042275:Y	NM_015363	ZIM2	intron	342
chr19:62043863:Y	NR_024059	MIMT1	upstream	56
chr19:62043863:Y	NM_006210	PEG3	exon	53
chr19:62043863:Y	NM_015363	ZIM2	exon	64
chr19:63247744:S	NM_182572	ZSCAN1	intron	309
chr19:63559937:K	NM_198458	ZNF497	exon	294
chr2:8738035:M	NM_002166	ID2	upstream	1338
chr2:24950576:S	NM_004036	ADCY3	intron	337
chr2:28621854:R	NM_153021	PLB1	intron	248
chr2:63505500:R	NM_015910	C2orf86	intron	233
chr2:63505500:R	NM_001042692	C2orf86	intron	233
chr2:71059484:R	NM_024933	ANKRD53	exon,intron	273
chr2:71059484:R	NM_001115116	ANKRD53	exon,intron	273
chr2:94899427:R	NM_144705	TEKT4	upstream	1233
chr2:94903947:M	NM_144705	TEKT4	intron	278
chr2:100951327:Y	NM_002518	NPAS2	exon,intron	106
chr2:109113174:W	NM_001099289	SH3RF3	intron	184
chr2:132727352:M	NR_027020	NCRNA00164	intron	329
chr2:132728084:R	NR_027020	NCRNA00164	intron	364
chr2:132730409:M	NR_027020	NCRNA00164	intron	220
chr2:132731578:R	NR_027020	NCRNA00164	exon,intron	320
chr2:218895611:Y	NM_022572	PNKD	upstream	455
chr2:218895611:Y	NM_015488	PNKD	intron	169
chr2:236284095:R	NM_001037131	AGAP1	intron	256
chr2:236284095:R	NM_014914	AGAP1	intron	256

chr2:238479562:M	NM_005855	RAMP1	intron	286
chr2:239649115:Y	NM_006037	HDAC4	intron	293
chr2:239932430:Y	NM_006037	HDAC4	intron	261
chr2:241356473:Y	NM_004321	KIF1A	intron	125
chr2:241699664:M	NM_015148	PASK	intron	285
chr2:241706887:R	NM_015148	PASK	intron	231
chr2:242083482:K	NM_006374	STK25	exon	244
chr20:4090809:R	NM_175840	SMOX	intron	216
chr20:4090809:R	NM_175841	SMOX	intron	216
chr20:4090809:R	NM_175842	SMOX	intron	216
chr20:4090809:R	NM_175839	SMOX	intron	216
chr20:25696285:R	NR_027061	MGC51338	intron	820
chr20:56849390:R	NM_016592	GNAS	exon,intron	279
chr20:56849390:R	NR_002785	GNASAS	intron	279
chr20:56851466:Y	NM_016592	GNAS	intron	321
chr20:56851466:Y	NR_002785	GNASAS	intron	321
chr20:56860527:Y	NM_080425	GNAS	upstream	853
chr20:56860527:Y	NM_001077490	GNAS	upstream	853
chr20:56860527:Y	NM_016592	GNAS	intron	196
chr20:56860527:Y	NR_002785	GNASAS	upstream	1027
chr20:56863963:W	NM_001077490	GNAS	intron	179
chr20:56863963:W	NM_080425	GNAS	intron	179
chr20:56863963:W	NM_016592	GNAS	intron	179
chr20:56896867:S	NR_003259	GNAS	upstream	504
chr20:56896867:S	NM_001077490	GNAS	intron	368
chr20:56896867:S	NM_080425	GNAS	intron	368
chr20:56896867:S	NM_016592	GNAS	intron	368
chr20:59337797:Y	NM_001794	CDH4	intron	249
chr20:60990433:R	NM_080797	DIDO1	exon	253
chr20:60990433:R	NM_033081	DIDO1	intron	253
chr20:62329137:S	NM_004535	MYT1	exon,intron	311
chr21:14274743:Y	NR_027270	C21orf81	exon	29
chr21:32167598:S	NM_014586	HUNK	exon	254
chr21:38551067:R	NM_170736	KCNJ15	exon,intron	259
chr21:38551067:R	NM_002243	KCNJ15	exon,intron	259
chr21:42192603:M	NM_015500	C2CD2	exon,intron	368
chr21:42192603:M	NM_199050	C2CD2	exon,intron	368
chr21:44530416:Y	NM_000383	AIRE	exon,intron	187
chr21:45468901:R	NM_001145407	ADARB1	intron	264
chr21:45468901:R	NM_015833	ADARB1	intron	264
chr21:45468901:R	NM_001033049	ADARB1	intron	264
chr21:45468901:R	NM_001112	ADARB1	exon	264
chr21:45468901:R	NM_015834	ADARB1	intron	264

chr22:19109604:K	NM_153334	SCARF2	exon	157
chr22:19109604:K	NM_182895	SCARF2	exon	157
chr22:20622217:Y	NM_014634	PPM1F	intron	290
chr22:22464394:Y	NM_001007468	SMARCB1	intron	261
chr22:22464394:Y	NM_003073	SMARCB1	intron	261
chr22:27405694:S	NM_001145418	TTC28	exon	219
chr22:28611138:R	NM_021090	MTMR3	intron	654
chr22:28611138:R	NM_153051	MTMR3	intron	654
chr22:28611138:R	NM_153050	MTMR3	intron	654
chr22:45238564:R	NM_014246	CELSR1	exon	211
chr22:45576719:R	NM_014346	TBC1D22A	intron	286
chr22:47435334:R	NM_015381	FAM19A5	intron	98
chr22:47435334:R	NM_001082967	FAM19A5	intron	98
chr22:48412318:R	NR_026997	C22orf34	intron	207
chr22:48436472:R	NR_026997	C22orf34	exon	288
chr22:48815745:R	NM_001080447	TTLL8	intron	449
chr22:49244892:R	NM_002972	SBF1	exon	112
chr3:16221473:Y	NM_054110	GALNTL2	intron	121
chr3:75876258:R	NM_001128223	ZNF717	intron	609
chr3:140145697:R	NM_023067	FOXL2	exon	91
chr3:196974738:S	NM_004532	MUC4	exon,intron	276
chr3:196974738:S	NM_018406	MUC4	exon,intron	276
chr3:196974738:S	NM_138297	MUC4	exon,intron	276
chr3:197019179:M	NM_004532	MUC4	intron	276
chr3:197019179:M	NM_018406	MUC4	intron	276
chr3:197019179:M	NM_138297	MUC4	intron	276
chr4:1210862:Y	NM_001012614	CTBP1	intron	259
chr4:1210862:Y	NM_001328	CTBP1	intron	259
chr4:2273521:S	NM_020972	ZFYVE28	intron	149
chr4:2388243:S	NM_020972	ZFYVE28	intron	295
chr4:4278909:R	NM_177998	OTOP1	exon,intron	448
chr4:4279357:K	NM_177998	OTOP1	exon	294
chr4:7430406:M	NM_020777	SORCS2	intron	331
chr4:40913388:Y	NM_173075	APBB2	upstream	1931
chr4:113705494:M	NM_001099776	LOC91431	intron	280
chr4:152549944:S	NM_001109977	FAM160A1	exon,intron	256
chr4:170006774:R	NM_016081	PALLD	intron	271
chr4:184048481:R	NM_001012732	DCTD	exon	301
chr4:184048481:R	NM_001921	DCTD	exon	301
chr4:191098899:R	NM_004477	FRG1	exon	252
chr5:1140868:Y	NM_006598	SLC12A7	intron	213
chr5:1647879:S	NR_003263	SDHAP3	upstream	80
chr5:7902989:S	NM_001089584	C5orf49	intron	319

chr5:9752519:Y	NR_027112	LOC285692	intron	257
chr5:75612211:S	NM_014979	SV2C	intron	179
chr5:118719592:M	NM_014350	TNFAIP8	exon	166
chr5:118719592:M	NM_001077654	TNFAIP8	intron	185
chr5:134287366:R	NM_032151	PCBD2	intron	554
chr5:134287698:Y	NM_032151	PCBD2	intron	257
chr5:134288288:R	NM_032151	PCBD2	intron	374
chr5:134288729:R	NM_032151	PCBD2	intron	352
chr5:134288945:R	NM_032151	PCBD2	intron	383
chr5:134289274:R	NM_032151	PCBD2	intron	355
chr5:134290558:R	NM_032151	PCBD2	intron	436
chr5:134290858:R	NM_032151	PCBD2	intron	437
chr5:134291008:Y	NM_032151	PCBD2	intron	534
chr5:134291300:R	NM_032151	PCBD2	intron	383
chr5:134291910:R	NM_032151	PCBD2	intron	420
chr5:178383601:Y	NM_001136116	DKFZp686E2433	intron	586
chr5:178919042:R	NM_001040451	RUFY1	upstream	133
chr5:178919042:R	NM_001040452	RUFY1	upstream	133
chr5:178919042:R	NM_025158	RUFY1	intron	273
chr5:178919238:M	NM_001040451	RUFY1	exon	18
chr5:178919238:M	NM_001040452	RUFY1	exon	18
chr5:178919238:M	NM_025158	RUFY1	intron	266
chr5:179978950:S	NM_002020	FLT4	exon,intron	254
chr5:179978950:S	NM_182925	FLT4	exon,intron	254
chr6:236744:S	NM_020185	DUSP22	upstream	203
chr6:238274:M	NM_020185	DUSP22	intron	301
chr6:261343:R	NM_020185	DUSP22	intron	310
chr6:6559333:M	NM_004271	LY86	intron	317
chr6:6559333:M	NR_026970	LOC285780	intron	317
chr6:29756098:R	NM_001109809	ZFP57	intron	256
chr6:29756543:Y	NM_001109809	ZFP57	intron	279
chr6:29904285:R	NM_002127	HLA-G	exon,intron	258
chr6:30001557:R	NR_001317	HCG4P6	upstream	60
chr6:30019298:K	NM_002116	HLA-A	exon,intron	165
chr6:30019298:K	NM_001080840	HLA-A29.1	exon,intron	165
chr6:30052347:Y	NM_005844	HCG9	intron	224
chr6:30052347:Y	NM_001080840	HLA-A29.1	intron	224
chr6:30147077:Y	NM_025236	RNF39	exon	174
chr6:30147077:Y	NM_170769	RNF39	exon,intron	174
chr6:31663371:M	NM_205837	LST1	intron	320
chr6:31663371:M	NM_205840	LST1	intron	320
chr6:31663371:M	NM_007161	LST1	exon,intron	320
chr6:31663371:M	NM_205839	LST1	intron	320

chr6:31663371:M	NM_205838	LST1	intron	320
chr6:32836871:R	NR_003937	HLA-DQB2	intron	296
chr6:32836920:R	NR_003937	HLA-DQB2	intron	210
chr6:32836924:Y	NR_003937	HLA-DQB2	intron	237
chr6:36845477:R	NM_020939	CPNE5	intron	183
chr6:42152923:R	NM_138572	TAF8	exon,intron	217
chr6:136652565:M	NM_014739	BCLAF1	exon,intron	257
chr6:136652565:M	NM_001077441	BCLAF1	exon,intron	257
chr6:136652565:M	NM_001077440	BCLAF1	exon,intron	257
chr6:136652610:Y	NM_014739	BCLAF1	exon,intron	148
chr6:136652610:Y	NM_001077441	BCLAF1	exon,intron	148
chr6:136652610:Y	NM_001077440	BCLAF1	exon,intron	148
chr6:150112222:S	NM_005389	PCMT1	upstream	286
chr6:158790069:R	NM_020245	TULP4	exon,intron	294
chr6:158790069:R	NM_001007466	TULP4	exon,intron	294
chr6:158985741:S	NM_006519	DYNLT1	exon,intron	41
chr6:166780986:R	NM_001006932	RPS6KA2	intron	197
chr6:166780986:R	NM_021135	RPS6KA2	intron	197
chr7:576303:R	NM_002735	PRKAR1B	intron	173
chr7:592504:Y	NM_002735	PRKAR1B	intron	264
chr7:1589301:S	NR_027329	KIAA1908	exon,intron	317
chr7:1589301:S	NR_027328	KIAA1908	intron	317
chr7:1589301:S	NR_021487	KIAA1908	exon,intron	317
chr7:2609626:R	NM_152558	IQCE	intron	267
chr7:2609626:R	NM_001100390	IQCE	intron	267
chr7:19122981:S	NM_000474	TWIST1	exon	330
chr7:48934541:Y	NR_003595	CDC14C	upstream	58
chr7:56054813:K	NM_004577	PSPH	exon,intron	424
chr7:102874071:K	NM_198999	SLC26A5	upstream	105
chr7:102874071:K	NM_206885	SLC26A5	upstream	105
chr7:102874071:K	NM_206883	SLC26A5	upstream	105
chr7:102874071:K	NM_206884	SLC26A5	upstream	105
chr7:135274877:S	NM_145808	MTPN	intron	277
chr7:135274877:S	NM_001128619	LUZP6	intron	277
chr7:139772308:R	NM_001008749	RAB19	exon	249
chr7:153522774:Y	NM_130797	DPP6	intron	312
chr7:153522774:Y	NM_001039350	DPP6	intron	312
chr7:157996235:R	NM_130842	PTPRN2	intron	282
chr7:157996235:R	NM_130843	PTPRN2	intron	282
chr7:157996235:R	NM_002847	PTPRN2	intron	282
chr7:158603416:Y	NM_003382	VIPR2	intron	331
chr8:169693:K	NR_003572	RPL23AP53	intron	133
chr8:1630014:K	NM_004745	DLGAP2	intron	179

chr8:1637157:R	NM_004745	DLGAP2	exon	242
chr8:1641097:R	NM_004745	DLGAP2	exon	331
chr8:2053369:M	NM_003970	MYOM2	intron	286
chr8:2872872:R	NM_033225	CSMD1	intron	284
chr8:11743990:K	NM_001908	CTSB	exon,intron	186
chr8:11743990:K	NM_147781	CTSB	exon,intron	186
chr8:11743990:K	NM_147782	CTSB	exon,intron	186
chr8:11743990:K	NM_147783	CTSB	exon,intron	186
chr8:11743990:K	NM_147780	CTSB	exon,intron	186
chr8:101246933:M	NM_003114	SPAG1	intron	302
chr8:101246933:M	NM_172218	SPAG1	intron	302
chr8:128958553:M	NR_003367	PVT1	intron	166
chr8:142230214:M	NM_014957	DENND3	exon,intron	283
chr9:34642330:R	NM_004512	IL11RA	upstream	1424
chr9:34642330:R	NM_147162	IL11RA	upstream	1424
chr9:34642330:R	NM_001142784	IL11RA	exon,intron	315
chr9:95469224:R	NM_005392	PHF2	exon,intron	346
chr9:97278179:R	NM_001083607	PTCH1	exon,intron	326
chr9:97278179:R	NM_000264	PTCH1	exon,intron	326
chr9:97278179:R	NM_001083604	PTCH1	exon,intron	326
chr9:97278179:R	NM_001083603	PTCH1	exon,intron	326
chr9:97278179:R	NM_001083602	PTCH1	exon,intron	326
chr9:97278179:R	NM_001083605	PTCH1	exon,intron	326
chr9:97278179:R	NM_001083606	PTCH1	exon,intron	326
chr9:128224283:R	NM_033446	FAM125B	intron	208
chr9:129266518:W	NM_001005374	LRSAM1	intron	251
chr9:129266518:W	NM_138361	LRSAM1	intron	251
chr9:129266518:W	NM_001005373	LRSAM1	intron	251
chr9:129871150:R	NM_001006641	SLC25A25	intron	227
chr9:129871150:R	NM_197956	NAIF1	upstream	1665
chr9:135074989:Y	NM_014581	OBP2B	upstream	449
chr9:138835832:S	NM_017995	C9orf86	intron	206
chr9:138835832:S	NM_024718	C9orf86	intron	206
