# Supplementary material for: The DNA Methylome of Human Peripheral Blood Mononuclear Cells
Source: PLoS Biol. 2010 Nov 9;8(11):e1000533. doi: 10.1371/journal.pbio.1000533 (PMC2976721; doi:10.1371/journal.pbio.1000533)
Supplement: Table S6 — Validation of expression level of both alleles for genes displaying allele-specific methylation. (0.01 MB PDF) [file pbio.1000533.s018.pdf]

Table S6. Validation of expression level of both alleles for genes displaying allele-specific methylation.

Accession numbe	Gene	SNP pos	Allele 1	Allele 2	Methyl% of allele 1	Methyl% of allele 2	Count of allele1	Cout of allele2	Fold difference
NM_023011	UPF3A	992	G	A	11.27	64.1	17	4	4.25
NM_001077440	BCLAF1	1906	T	A	100	0	30	0	>10
NM_001256	CDC27	1350	G	A	17.24	85.88	22	3	7.33
NM_001256	CDC27	1561	C	A	17.24	85.88	22	3	7.33
NM_020185	DUSP22	1066	T	A	60	0	30	0	>10
NM_175911	OR2L13	1678	C	T	7.26	92.31	17	11	1.55
NM_052867	NALCN	6424	T	C	4.35	78.57	13	11	1.18
