# Supplementary material for: The DNA Methylome of Human Peripheral Blood Mononuclear Cells
Source: PLoS Biol. 2010 Nov 9;8(11):e1000533. doi: 10.1371/journal.pbio.1000533 (PMC2976721; doi:10.1371/journal.pbio.1000533)
Supplement: Table S7 — GO classification of allele-specific methylated genes. (0.06 MB PDF) [file pbio.1000533.s019.pdf]

Table S7. GO classification of allele-specific methylated genes.

GO category	# of genes	# of allele-specific methylated genes	P-value	Over/under-represent	# of heterozygotes (TSS +/- 2k)	P-value	heterozygous level comparing to average	Accessions	Gene names
regulation of cell shape	9	54	1.56E-12	Over	57	0.025013	more	NM_007161,NM_198999,NM_205837,NM_205838,NM_205839,NM_205840,NM_206883, NM_206884,NM_206885	LST1,SLC26A5
negative regulation of lymphocyte proliferation	5	5	1.14E-10	Over	21	0.530855	more	NM_007161,NM_205837,NM_205838,NM_205839,NM_205840	LST1
dendrite development	5	6	2.08E-10	Over	21	0.530855	more	NM_007161,NM_205837,NM_205838,NM_205839,NM_205840	LST1
cell morphogenesis	5	11	1.94E-09	Over	21	0.530855	more	NM_007161,NM_205837,NM_205838,NM_205839,NM_205840	LST1
secondary active sulfate transmembrane transporter activity	4	16	6.08E-07	Over	36	0.028165	more	NM_198999,NM_206883,NM_206884,NM_206885	SLC26A5
sulfate transport	4	22	1.84E-06	Over	36	0.028165	more	NM_198999,NM_206883,NM_206884,NM_206885	SLC26A5
guanyl nucleotide binding	3	7	4.47E-06	Over	15	0.432582	more	NM_001077490,NM_016592, NM_080425	GNAS

G-protein signaling, adenylate cyclase activating pathway	3	8	6.13E-06	Over	15	0.432582	more	NM_001077490,NM_016592, NM_080425	GNAS
motor activity	5	87	1.75E-05	Over	40	0.029222	more	NM_001080467,NM_198999, NM_206883,NM_206884,NM_206885	MYO5B,SLC26A5
phospholipid dephosphorylation	3	20	6.38E-05	Over	3	0.195293	less	NM_021090,NM_153050,NM_153051	MTMR3
protein secretion	3	28	0.000159	Over	15	0.432582	more	NM_001077490,NM_016592, NM_080425	GNAS
sister chromatid cohesion	2	4	0.000166	Over	10	0.74389	more	NM_001048205,NM_005132	REC8
inositol or phosphatidylinositol phosphatase activity	3	29	0.000175	Over	3	0.195293	less	NM_021090,NM_153050,NM_153051	MTMR3
heterotrimeric G-protein complex	3	37	0.000341	Over	15	0.432582	more	NM_001077490,NM_016592, NM_080425	GNAS
transcription repressor activity	4	94	0.000365	Over	16	0.679309	more	NM_001077440,NM_001077441,NM_002166,NM_014739	BCLAF1,ID2
protein serine/threonine phosphatase activity	3	52	0.000871	Over	3	0.195293	less	NM_021090,NM_153050,NM_153051	MTMR3
Golgi apparatus	5	208	0.000872	Over	21	0.530855	more	NM_007161,NM_205837,NM_205838,NM_205839,NM_205840	LST1
negative regulation of transcription	4	120	0.000881	Over	16	0.679309	more	NM_001077440,NM_001077441,NM_002166,NM_014739	BCLAF1,ID2

nucleocytoplasmic transporter activity	2	13	0.001135	Over	14	0.265473	more	NM_023011,NM_080687	UPF3A
female pregnancy	3	65	0.001609	Over	15	0.432582	more	NM_001077490,NM_016592, NM_080425	GNAS
cytoplasm	15	1867	0.002348	Over	55	0.400425	more	NM_000383,NM_001077490, NM_001256,NM_007161,NM_016592,NM_021090,NM_023011,NM_080425,NM_080687, NM_153050,NM_153051,NM_205837,NM_205838,NM_205839,NM_205840	AIRE,GNAS,CDC27,LST1,MTMR3,UPF3A
protein tyrosine phosphatase activity	4	164	0.002666	Over	12	1	more	NM_020185,NM_021090,NM_153050,NM_153051	DUSP22, MTMR3
inward rectifier potassium channel activity	2	21	0.002687	Over	8	1	more	NM_002243,NM_170736	KCNJ15
nuclear chromosome	2	21	0.002687	Over	10	0.74389	more	NM_001048205,NM_005132	REC8
transport	8	697	0.003369	Over	56	0.01602	more	NM_001006641,NM_014581, NM_023011,NM_080687,NM_198999,NM_206883,NM_206884,NM_206885	SLC25A25,OBP2B, UPF3A,SLC26A5

mRNA catabolic process, nonsense-mediated decay	2	25	0.003694	Over	14	0.265473	more	NM_023011,NM_080687	UPF3A
antigen processing and presentation of endogenous antigen	2	25	0.003694	Over	125	3.44E-10	more	NM_002116,NM_002127	HLA-A,HLA-G
sensory perception of sound	4	192	0.004601	Over	36	0.028165	more	NM_198999,NM_206883,NM_206884,NM_206885	SLC26A5
MHC class I protein complex	2	30	0.005161	Over	125	3.44E-10	more	NM_002116,NM_002127	HLA-A,HLA-G
antigen processing and presentation of endogenous peptide antigen via MHC class I	2	33	0.00615	Over	125	3.44E-10	more	NM_002116,NM_002127	HLA-A,HLA-G
meiotic recombination	2	33	0.00615	Over	10	0.74389	more	NM_001048205,NM_005132	REC8
hematopoietin/interferon-class (D200-domain) cytokine receptor activity	2	34	0.006497	Over	2	0.288737	less	NM_004512,NM_147162	IL11RA
chromosome organization and biogenesis	3	109	0.006541	Over	18	0.303638	more	NM_000474,NM_001048205, NM_005132	TWIST1, REC8
mitotic metaphase/anaphase transition	1	1	0.006616	Over	1	0.461028	less	NM_001256	CDC27
MHC class I receptor activity	2	36	0.007217	Over	125	3.44E-10	more	NM_002116,NM_002127	HLA-A,HLA-G

skeletal development	3	115	0.007545	Over	24	0.109163	more	NM_000474,NM_001007139, NM_005220	TWIST1,IGF2,DLX3
antigen processing and presentation	2	38	0.007972	Over	125	3.44E-10	more	NM_002116,NM_002127	HLA-A,HLA-G
protein amino acid dephosphorylation	4	235	0.009103	Over	12	1	more	NM_020185,NM_021090,NM_153050,NM_153051	DUSP22,MTMR3
aspartoacylase activity	1	2	0.009908	Over	13	0.132352	more	NM_080658	ACY3
prothoracicotrophic hormone activity	1	3	0.013188	Over	8	0.460406	more	NM_001007139	IGF2
protein-L-isoaspartate (D-aspartate) O-methyltransferase activity	1	3	0.013188	Over	6	0.68346	more	NM_005389	PCMT1
odorant binding	1	3	0.013188	Over	2	1	less	NM_014581	OBP2B
meiosis	2	55	0.015702	Over	10	0.74389	more	NM_001048205,NM_005132	REC8
chemosensory behavior	1	4	0.016458	Over	2	1	less	NM_014581	OBP2B
transporter activity	5	437	0.017944	Over	38	0.040647	more	NM_014581,NM_198999,NM_206883,NM_206884,NM_206885	OBP2B,SLC26A5
mRNA export from nucleus	2	64	0.020684	Over	14	0.265473	more	NM_023011,NM_080687	UPF3A
induction of apoptosis	3	173	0.021822	Over	12	0.77231	more	NM_001077440,NM_001077441,NM_014739	BCLAF1

immune response	5	466	0.022774	Over	21	0.530855	more	NM_007161,NM_205837,NM_205838,NM_205839,NM_205840	LST1
negative regulation of S phase of mitotic cell cycle	1	6	0.022964	Over	3	1	more	NM_173075	APBB2
cytoplasmic dynein complex	1	6	0.022964	Over	2	1	less	NM_006519	DYNLT1
voltage-gated ion channel activity	2	68	0.023081	Over	8	1	more	NM_002243,NM_170736	KCNJ15
