# Supplementary material for: The DNA Methylome of Human Peripheral Blood Mononuclear Cells
Source: PLoS Biol. 2010 Nov 9;8(11):e1000533. doi: 10.1371/journal.pbio.1000533 (PMC2976721; doi:10.1371/journal.pbio.1000533)
Supplement: Table S8 — Validation of methylation level of CpG by Sanger sequencing. (0.03 MB PDF) [file pbio.1000533.s020.pdf]

Table S8. Validation of methylation level of CpG by Sanger sequencing.

Chr	position	GA sequencing		Sanger validation	
		methylated	total	methylated	total
chr1	114854255	0	22	1	6
chr1	114854325	0	13	0	7
chr1	114854333	0	15	0	7
chr1	114854352	0	10	0	7
chr1	114854365	0	11	0	7
chr5	118719538	2	7	0	22
chr5	118719546	2	4	0	22
chr5	118719551	2	4	0	22
chr5	118719593	2	9	1	21
chr5	118719596	0	10	1	21
chr5	118719602	2	7	1	21
chr5	118719609	0	8	0	22
chr5	118719619	1	7	0	22
chr5	118719627	1	8	1	21
chr5	118719632	0	7	0	22
chr5	118719636	1	6	1	21
chr5	118719650	1	5	1	21
chr5	118719657	0	6	1	21
chr5	178918949	4	11	4	5
chr5	178918978	7	7	4	5
chr5	178919006	5	8	5	4
chr5	178919010	7	7	4	5
chr5	178919018	4	10	3	6
chr5	178919073	1	8	3	6
chr5	178919087	1	5	3	6
chr12	380735	2	11	2	26
chr12	380737	2	11	8	20
chr12	380785	0	9	0	28
chr12	380796	0	10	0	28
chr12	380811	0	12	0	28
chr12	380823	0	9	1	27
chr12	380837	0	11	0	28
chr12	380855	0	11	0	28
chr12	380858	0	12	0	28
chr12	380863	1	10	0	28
chr13	45525428	1	13	2	23
chr13	45525473	3	19	2	23
chr13	45525476	6	15	4	21
chr13	45525504	5	24	1	24
chr13	45525507	6	22	1	24
chr15	49700242	9	9	18	5
chr15	49700249	9	9	16	7
chr16	88250809	0	4	3	21
chr16	88250816	0	4	2	22
chr17	1477466	1	9	0	25
chr17	1477471	2	9	1	24
chr17	1477534	2	8	2	23
chr17	1477542	5	7	11	14
chr17	1477554	4	9	8	17
chr17	1477566	1	12	3	22
